# Single-pollen-cell sequencing for gamete-based phased diploid genome assembly in plants

**DOI:** 10.1101/gr.251033.119

**Published:** 2019-11

**Authors:** Dongqing Shi, Jun Wu, Haibao Tang, Hao Yin, Hongtao Wang, Ran Wang, Runze Wang, Ming Qian, Juyou Wu, Kaijie Qi, Zhihua Xie, Zhiwen Wang, Xiang Zhao, Shaoling Zhang

**Affiliations:** 1 Centre of Pear Engineering Technology Research, State Key Laboratory of Crop Genetics and Germplasm Enhancement, Nanjing Agricultural University, Nanjing 210095, China;; 2 Center for Genomics and Biotechnology, Fujian Agriculture and Forestry University, Fuzhou 350002, Fujian Province, China;; 3 School of Life Science, Henan University, Kaifeng 475004, China;; 4 College of Agriculture, Qingdao Agricultural University, Qingdao 266109, China;; 5 PubBio-Tech, Wuhan 430070, China

## Abstract

Genome assemblies from diploid organisms create mosaic sequences alternating between parental alleles, which can create erroneous gene models and other problems. In animals, a popular strategy to generate haploid genome-resolved assemblies has been the sampling of (haploid) gametes, and the advent of single-cell sequencing has further advanced such methods. However, several challenges for the isolation and amplification of DNA from plant gametes have limited such approaches in plants. Here, we combined a new approach for pollen protoplast isolation with a single-cell DNA amplification technique and then used a “barcoding” bioinformatics strategy to incorporate haploid-specific sequence data from 12 pollen cells, ultimately enabling the efficient and accurate phasing of the pear genome into its A and B haploid genomes. Beyond revealing that 8.12% of the genes in the pear reference genome feature mosaic assemblies and enabling a previously impossible analysis of allelic affects in pear gene expression, our new haploid genome assemblies provide high-resolution information about recombination during meiosis in pollen. Considering that outcrossing pear is an angiosperm species featuring very high heterozygosity, our method for rapidly phasing genome assemblies is potentially applicable to several yet-unsequenced outcrossing angiosperm species in nature.

With the emergence of high-throughput sequencing technologies, the draft genomes of many species have been released, but many genomes, particularly those that have high levels of heterozygosity or are polyploid, potentially contain many mosaic sequences because parental alleles are randomly selected or collapsed during genome assembly. This is problematic because certain haplotype features are very important in some analyses, for instance in linkage analysis or population genetics and functional studies (Koren et al. 2018; Yang et al. 2019). Without an accurate allele-level reference, identification of variation between homologous chromosomes, allele-specific expression, and haplotype-specific features is challenging (Hoehe et al. 2014; Church et al. 2015). To address this problem, advanced sequencing technologies coupled with bioinformatics techniques to phase individual alleles have been developed (Aguiar and Istrail 2012; Weisenfeld et al. 2017). Although some progress has been made in reconstructing haplotype-resolved human and animal genomes (Aguiar and Istrail 2012; Weisenfeld et al. 2017), haplotype-resolved genome assembly in plants is less developed, with a key limiting factor being the much higher level of heterozygosity in some outcrossing plant species (Chin et al. 2016).

Several experimental and computational methodologies have been developed to discriminate among two haplotypes in a diploid genome. For instance, breeding a doubled-haploid (DH) line may be the most straightforward method. However, breeding a DH line can be laborious and the probability of success is relatively low (Xu et al. 2013; Daccord et al. 2017). Another approach is to use microfluidics-based chromosomal isolation techniques (Fan et al. 2011) to directly separate each homologous chromosome, but the equipment and technical requirements are still prohibitive and there is perhaps still a long way to go before it can become widely adopted.

Sequencing technology combined with computational phasing algorithms (Chin et al. 2016; Yang et al. 2017) is much more accessible. This method has seen some use in diploid and polyploid genome assembly and could solve the problem of heterozygous assembly to some extent. Platanus (Kajitani et al. 2014), MaSuRCA (Zimin et al. 2013), and SOAPdenovo2 (Luo et al. 2012) have been used for short-read sequencing data assembly. Canu (Koren et al. 2017) and FALCON (Chin et al. 2016) were developed for assembling long-read sequences (e.g., BAC, fosmid, 10x Genomics, and single-molecule sequencing reads from Pacific Biosciences [PacBio] and Oxford Nanopore) and potentially can be used to resolve individual haplotypes. However, all computer algorithms are naturally limited by the length of the reads that carry the haplotype phasing information; in many cases, they can only randomly select heterozygous reads but not accurately determine which set of heterozygous reads belongs to which haplotype, leading to switching errors (Church et al. 2015; Chin et al. 2016). Both problems mainly result from the absence of long-range, preferably chromosome-scale haplotype information. However, such haplotype information is readily available in some haploid cell lines, such as sperm or pollen, with minimal switching between haplotypes only attributable to meiotic recombination (Kirkness et al. 2013).

In recent years, the biological significance of allelic gene expression in plant growth and epigenetic regulation has been studied and is now better understood (He et al. 2010; Reinius and Sandberg 2015). For example, loss of function of the *SEMI DWARF 1* (*SD1*) allelic gene led to the “green revolution” in Asia, and seven *sd1* alleles have been used in breeding of semidwarf rice varieties in China, the United States, and Japan. The *Arabidopsis FLOWERING LOCUS T* (*FT*) gene and its orthologs in other plant species participate in plant flowering. Allelic variation of the *FT* gene in perennial ryegrass is correlated with variation in flowering time (Skøt et al. 2011). Furthermore, allelic *MYB* transcription factors in fruit trees have been shown to differentially control anthocyanin biosynthesis and fruit color (Lin-Wang et al. 2010; Zhu et al. 2011). Accurately haplotype-based allele mining is a prerequisite for the precise characterization of allelic variation in genes controlling agronomic traits.

In our previous study in 2013, we conducted long-read sequencing (BAC-seq) and assembled an initial draft of the pear genome (Wu et al. 2013); however, we were unable to assemble haploid-resolved genomes using the available phasing algorithms on account of two challenges: On the one hand, pear has a very high heterozygosity (∼1.02%) and the parents of the species are unknown; on the other hand, the assembled BAC sequences had relatively low contiguity. Further, there have been previous unsuccessful attempts to generate DH pear using anther culture (Germanà 2011). We were thus motivated to use gamete sequencing to facilitate the phasing of the diploid pear genome into its two haploid genomes.

## Results

### Whole-genome amplification and sequencing of a single pollen cell

**Figure 1.**
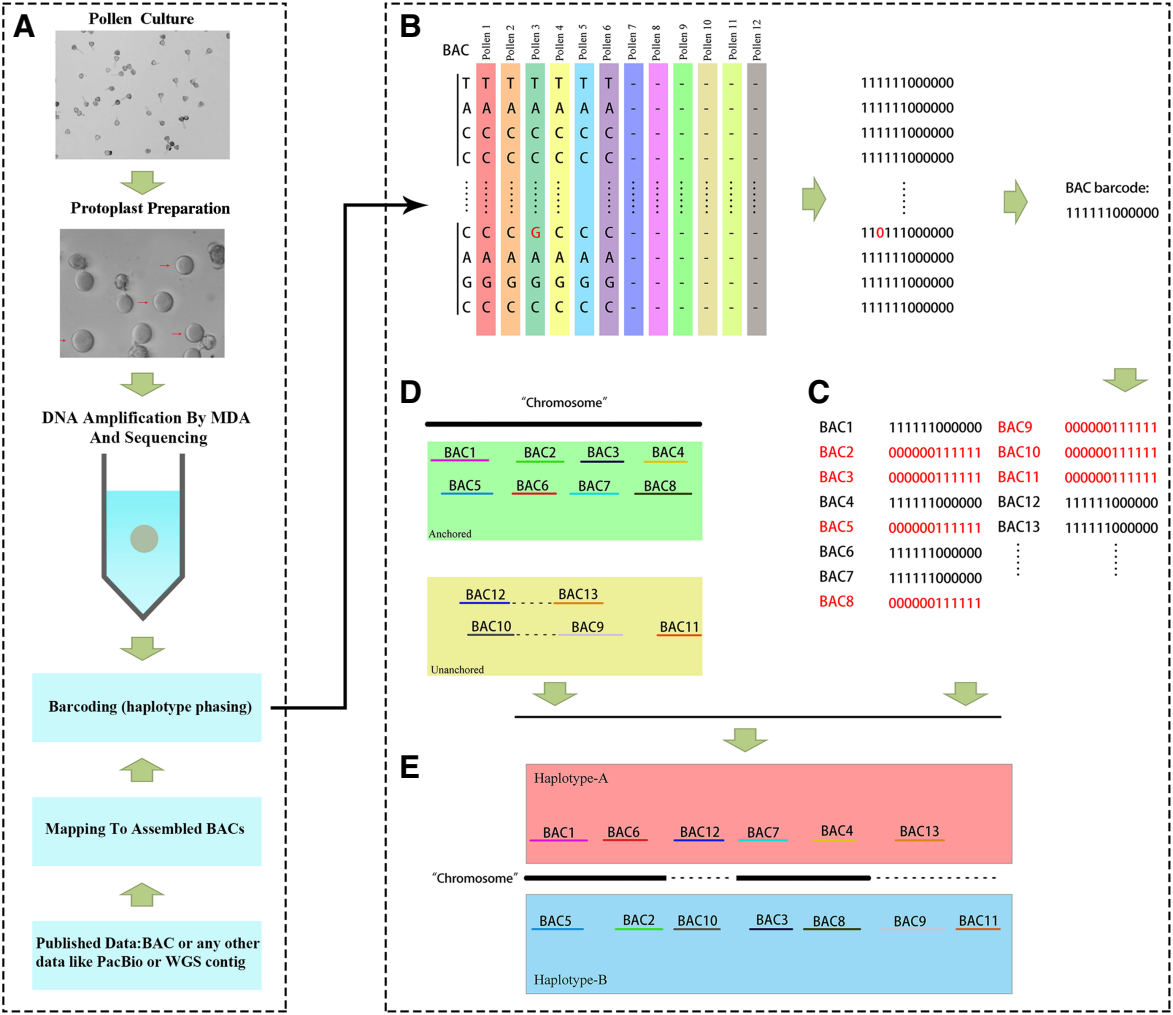
Schematic of haploid phasing using a barcoding approach. (*A*) Pollen is a haploid gamete. Here, we combined a new approach for pollen protoplast isolation with a single-cell DNA amplification technique and then used a barcoding bioinformatics strategy to incorporate haploid-specific sequence data from pollen cells, ultimately enabling the efficient and accurate phasing to assemble a haplotype genome as well as detecting meiotic recombination events. (*B*) Establishing the relationship between 12 pollen cells and BAC sequences using 12-bit binary codes in which “1” indicates identical and “0” indicates “not identical” or “absent.” Each base was labeled with a 12-bit binary code. Finally, each BAC was labeled with a specific 12-bit binary code. (*C*) BACs from different haplotypes received different 12-bit binary codes. (*D*) The chromosomal location of each BAC on the reference genome chromosomes was determined by aligning the BACs to the anchored reference chromosomes. (*E*) BACs were classified into haplotypes based on the chromosomal location and the 12-bit binary code.

Because pollen is relatively easier to isolate than ovules, our experimental approach initially focused on isolating the haploid genomes of pollen grains (Fig. 1A). The cell walls of pollen tubes are quite fragile, and pollen nuclei (both the vegetative nucleus and the sperm nucleus) are transferred within elongating pollen tubes (Lu et al. 2015). We successfully adopted the strategy of first destroying the cell wall using cell wall degrading enzymes (cellulases and pectinases) and then using thin glass pipettes to obtain single pollen protoplasts. After lysing these single protoplasts, we used the multiple displacement amplification (MDA) method (Dean et al. 2001) to amplify the genomic DNA of each single pollen protoplast. The MDA method is known to sometimes generate template-independent products (in which exogenous DNA contamination is introduced during the amplification step) (Pan et al. 2008). To estimate if pear genome sequences are enriched in the amplified DNA product, sequences from 11 chromosomes from the reference genome were selected for comparisons (Supplemental Table S1; for details, see Methods), and 12 had amplified DNA of sufficient quality to be used for further high-throughput sequencing. To obtain the whole-genome sequence for each pollen cell, each MDA product was sequenced at 7.5- to 12-fold depth of reference genome coverage on the Illumina HiSeq 2000/4000 platform with 100/125 nt double paired ends. After removing adapter sequences as well as ambiguous and/or low-quality reads, a total of 0.98 billion reads covering an average of ∼66% of the pear reference genome were generated for each single pollen cell (the breadth of coverage of each pollen cell could be aligned to each chromosome). Collectively, these sequences covered 98.85% of the pear genome. The average mapping rate for reads from a single cell was ∼79.86% (with ∼81.24% unique loci) (Supplemental Table S2). To estimate if mutations caused by MDA (Dean et al. 2001) affected the haplotype phase, a total of 3,506,724 SNPs (1.2–1.9 million SNPs in each pollen) were identified across the 12 pollen cells using BWA (Supplemental Table S2; Li and Durbin 2009); hence, the frequency of SNPs was 1.05%, which is similar to the estimated heterozygosity (∼1.02%) reported previously (Wu et al. 2013). Thus, we had obtained enough sufficiently high-quality haplotype genome data from the 12 pollen cells to proceed with haplotype phasing of the pear genome.

### Phasing of the haploid genomes of diploid pear using a “barcoding” approach

We completely reassembled the haploid genomes of diploid pear based on the combination of existing BAC sequence data and the new single-pollen-cell sequence data. A total of 38,304 BACs from the initial pear genome assembly project are available (Wu et al. 2013), and these BACs were assembled individually using SOAPdenovo2 (N50 = 17 kb) (Luo et al. 2012). The chromosomal locations of all BACs that aligned to the reference genome with >80% breadth of coverage were retrieved; there were 25,127 anchored and 13,177 unanchored BACs (Fig. 1D). Each of the 12 pollen cell sequence read data sets was then aligned to each assembled BAC sequence, and SNPs from each pollen cell were called (Fig. 1B). In this step, the genotype for a given SNP position on a BAC must occur in at least two pollen cells. Then, each SNP position on the BAC was then summarized with a 12-bit binary code that contained a marker for the haplotype composition for each of the 12 pollen cells (Fig. 1C); that is, for a given SNP position on a BAC, if the nucleotide was the same as that of the BAC sequence, it was assigned as 1, if the nucleotide at this position was absent or differed from the BAC, it was assigned 0, and this was performed with the data for each of the 12 pollen cells.

Specifically, the 12-bit code, which is effectively a “barcode,” was then used to determine which of the haploid genomes the BAC was derived from. Specifically, if there was no recombination in any of the pollen cells, each chromosome should be uniformly encoded by a single 12-bit binary code. However, when we do consider the possibility of meiotic recombination, one haploid chromosome might contain several different 12-bit binary codes, because some pollen cells switch haplotypes during recombination. Therefore, the hamming distance (the hamming distance between two barcodes measures the number of differences required to change one barcode into another) between 12-bit codes was calculated and used to determine from which haploid genome a given BAC was derived. Using the BAC barcode information (Supplemental Table S3), a total of 31,312 BACs, representing 81.7% of the BACs, were phased as either A or B haploid genome by filtering by barcode type and barcode frequency. Additionally, most of the A:B ratio of phased BACs in each chromosome is close to 50:50 (Supplemental Table S4).

Phasing proceeded with the following prerequisites: First, the binary barcodes of the BACs from the same chromosome, but from different haploid genomes, would feature one or more complementary values at some SNP position (Fig. 1C). Second, owing to meiotic recombination, each haploid genome chromosome will contain several binary barcode types. Hence, the hamming distances between the binary barcodes of each BAC were calculated, and the closest were chosen as belonging to the same haplotype chromosome (Fig. 1E; Supplemental Table S3). Using these stringent criteria, we found that there were 6992 BACs (18.25%) that failed to phase (typically because genotypes were supported by sequencing data from only one pollen cell or the hamming distance was close to two or more chromosomes). Furthermore, we randomly selected 13G original WGS data, 196 phased BACs, and 188 unphased BACs for calculating WGS depth of coverage. We found the average depth of coverage for unphased BACs is about 1626 versus phased BACs, which is about 405.43 (Supplemental Table S10). Therefore, we deduced that such BACs may belong to multiple chromosomes in the pear genome owing to regions of high sequence similarity between chromosomes. Note that, seeking to achieve completeness during the previous draft release of the pear genome, these 6697 BACs were iteratively assembled into each chromosome based on the amount of overlap rather than based on the pollen barcode information.

Heterozygosity is known to affect the quality of a given genome assembly, because it induces bifurcating structures (or “bubbles”) as the assembly graphs are generated. Phasing of homologous chromosomes and thus reducing heterozygosity and correcting many of the mosaic and/or collapsed sequences should in theory substantially improve the quality of a genome assembly. We assembled a total of 34 chromosomes individually, using the corresponding phased BAC sequence reads and the 6697 initially unanchored BACs. The initial contig N50 values for haploid genome A and haploid genome B were on average 1.69 and 1.80 kb, respectively. To determine the preliminary quality of the BAC phasing, the chromosomes from each of the haploid genomes were merged into an assembly, and we found that these merged chromosomes had initial contig N50 values of 591 bp on average (Supplemental Table S5), showing that contig N50 values of haploid genome A and haploid genome B were 2.8–3 times longer than those for of the merged assembly, illustrating that the continuity of the assembly improved on phasing.

Finally, previously obtained whole-genome shotgun (WGS) mate-pair library sequence data (2, 5, 10, 20, and 40 kb) were used to build superscaffolds (Wu et al. 2013); BAC sequence reads (250 and 500 bp) were used again to fill in gaps, and the final scaffold N50s for haploid genome A and haploid genome B were on average 108 and 107 kb, respectively (Supplemental Table S6). The assembled genome sizes of haploid genome A and haploid genome B were 546 Mb (11,315 scaffolds) and 536 Mb (10,706 scaffolds), respectively, and the chromosome-anchored genome sizes were 382 and 374 Mb, representing 69.96% and 69.78% of the assembled genome size (Supplemental Table S7).

### Comparisons between the two haplotype genomes and the reference genome

Comparison of the two phased haploid genomes with the reference genome revealed differences in chromosome lengths, with for example Chromosome 1 of haploid genome A being 32 Mb, versus the ∼29 Mb length of haploid genome B and the ∼11 Mb Chromosome 1 of the reference genome (Supplemental Table S7). We used BUSCO v2 (Simão et al. 2015) to evaluate the assembly quality for haploid genomes A and B; in this analysis, a set of genes conserved in eukaryotes is used as a proxy to assess genic completeness. This analysis indicated that the two haploid genome assemblies are of higher quality than the reference genome (Table 1), with each haploid genome containing a higher number of complete BUSCO genes than the reference genome (90.5% vs. 89.8%). When the two haploid genomes were considered together, the merged genome, containing 95.2% of the expected BUSCO genes, was much more complete.

**Table 1.**
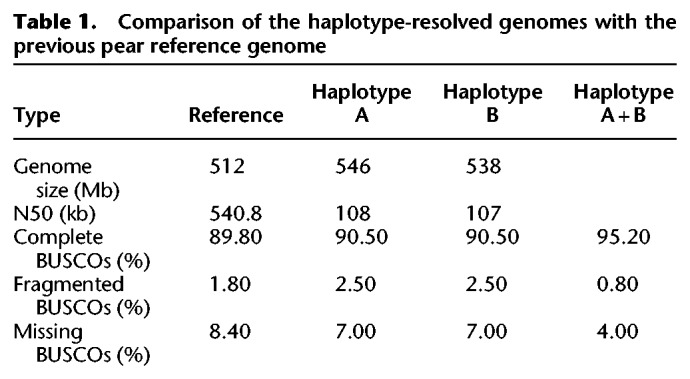
Comparison of the haplotype-resolved genomes with the previous pear reference genome

To further evaluate the quality of the haploid genome assemblies, genes were annotated using a similar set of parameters and methods as had been used for the reference genome, and a direct comparison was performed. In total, 41,904 genes (∼98% of those in the reference genome), including 37,805 in haploid genome A and 37,267 in haploid genome B, were annotated; and 33,559 genes were anchored in haploid genome A and 33,060 genes were anchored in haploid genome B (Table 2). Additionally, although we found that 1190 anchored genes in the reference genome assembly were absent in the anchored A and B haploid genomes, 6420 unanchored genes in the reference genome were anchored to the both haplotype genomes in this study, again highlighting the comprehensiveness of the phased assembly.

**Table 2.**
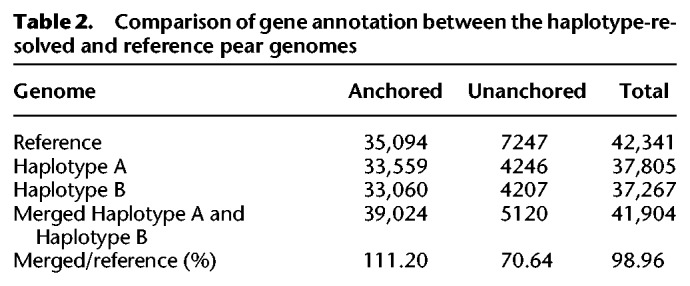
Comparison of gene annotation between the haplotype-resolved and reference pear genomes

### Mosaic assembly in the reference genome

**Figure 2.**
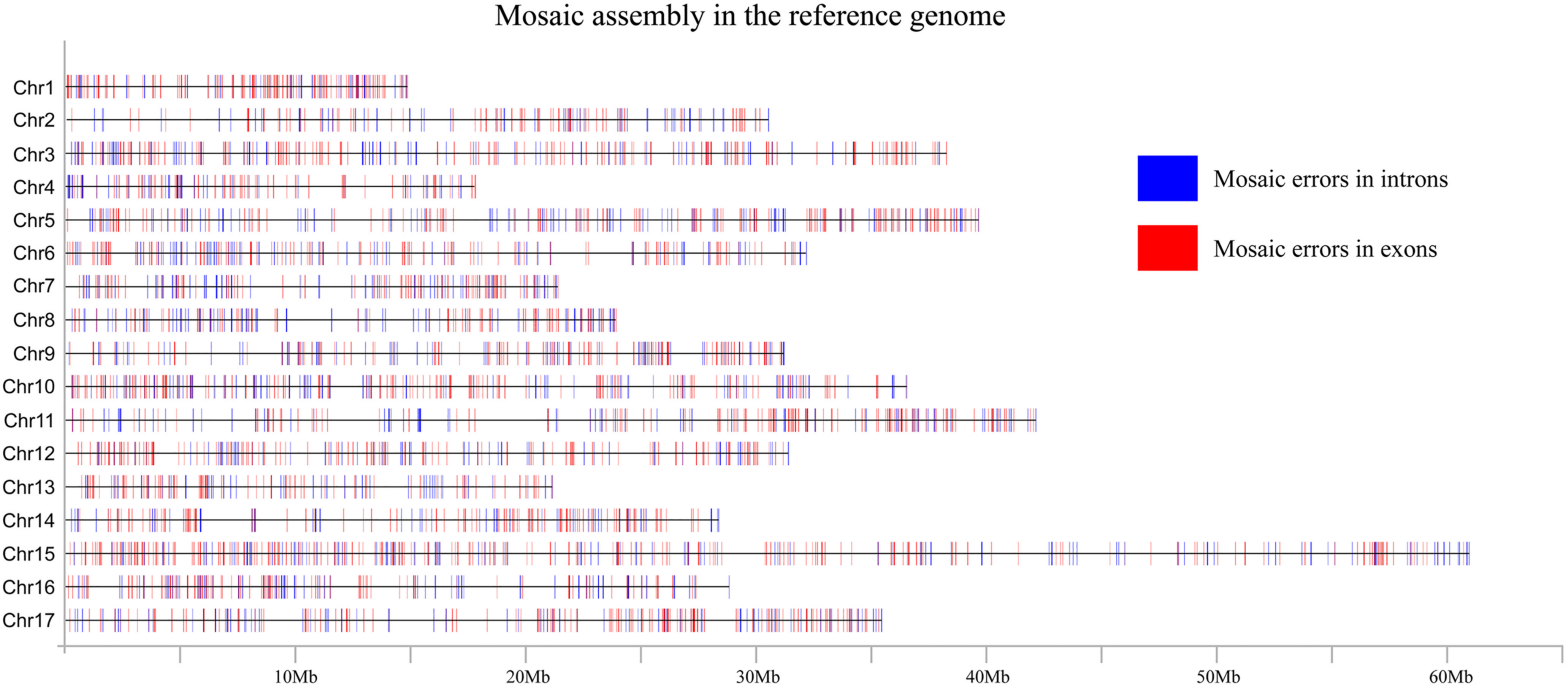
Distribution of mosaic sequences in genome assemblies built from incompletely phased data. Genes were classified as two types: mosaic errors in introns (blue) and mosaic errors in exons (red).

Recalling the well-recognized problem of mosaic sequences in genome assemblies built from incompletely phased data, it is exceedingly likely that sequences for multiple loci of the pear reference genome assembly are actually mosaic. Addressing this, we compared the sequences of the reference genome with the sequences for the same genes in haploid genome A and haploid genome B and identified 3479 genes (∼8.12%) in the reference genome that are apparently mosaic sequences (Fig. 2), including 2332 genes with mosaic errors in their exons and 1147 genes with mosaic errors in their introns. This finding strongly supports the need to adopt approaches like our gamete barcoding to phase genomes and thereby obtain more accurate and informative genome sequences for downstream functional studies.

**Figure 3.**
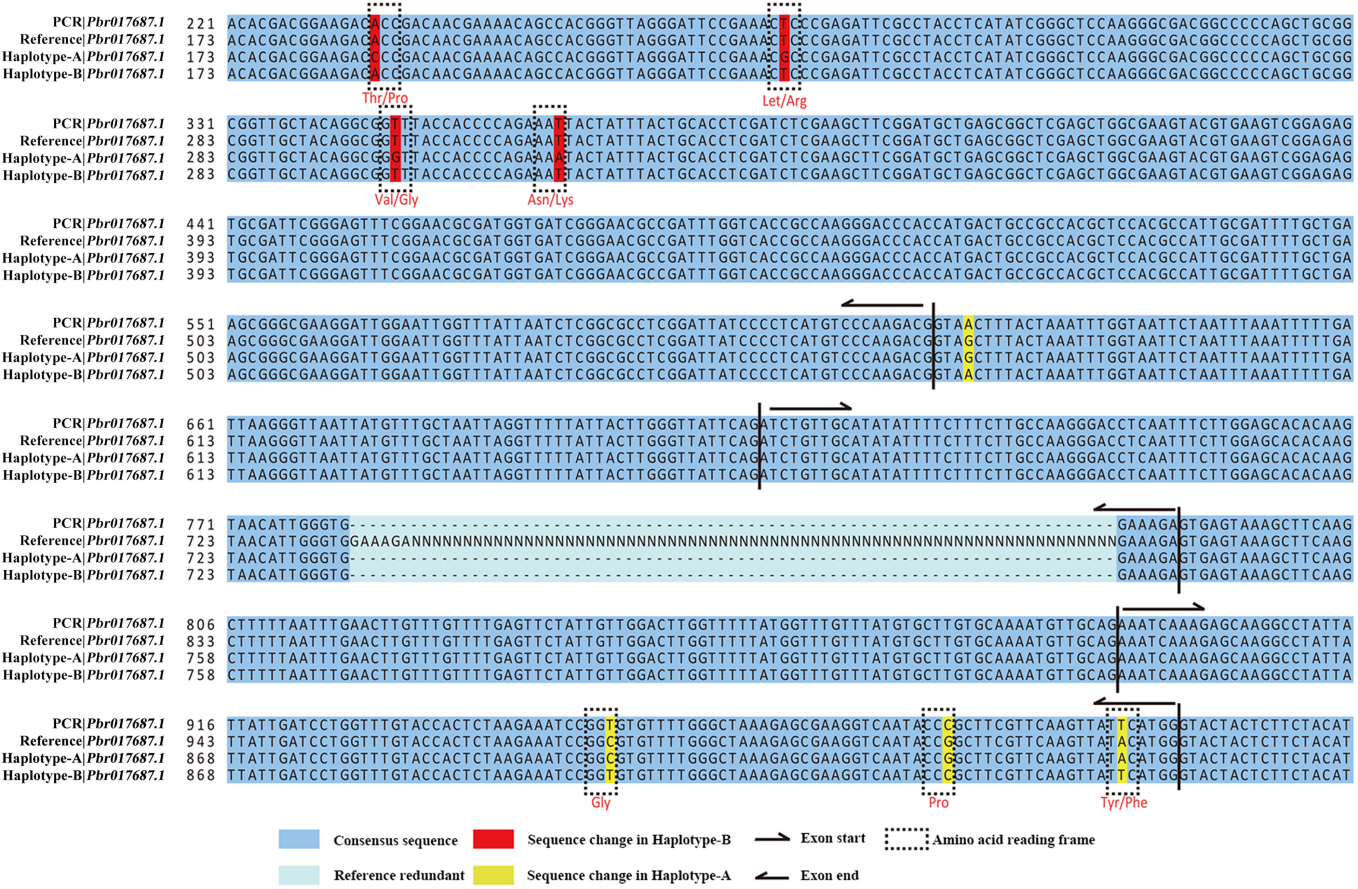
Mosaic assembly of *Pbr017687.1* in the reference genome. Four base pairs in yellow only matched haplotype A, and 4 bp in red only matched haplotype B. Five of these eight sequences result in differences in amino acid sequence. (N) Redundant sequences in the reference.

To validate the correctness of the phased haploid genome sequences over the mosaic sequence in the reference genome, 23 genes with suspected mosaic errors in the reference genome were randomly selected for Sanger sequencing (Fig. 3; Supplemental Table S8). We found that the sequences for 18 of the 23 genes were identical to either the A or B haploid genome sequence. In contrast, the sequences for two of the 23 genes were identical to the reference genome sequence, suggesting that there are some errors in the phased A and B haploid genome assemblies. Three of the 23 genes were not identical to the reference assembly or either of the haploid genome assemblies. Furthermore, we found that the problem of mosaic in *Pbr017687.1* (Fig. 3) in the initial reference genome is definitely the false overlaps of two BACs, which were from two haplotypes in Chr 12 but merged into the initial assembled reference genome. Hence, we suspect that these apparent errors might be related to false BAC-to-BAC overlaps in the initial reference genome assembly.

**Figure 4.**
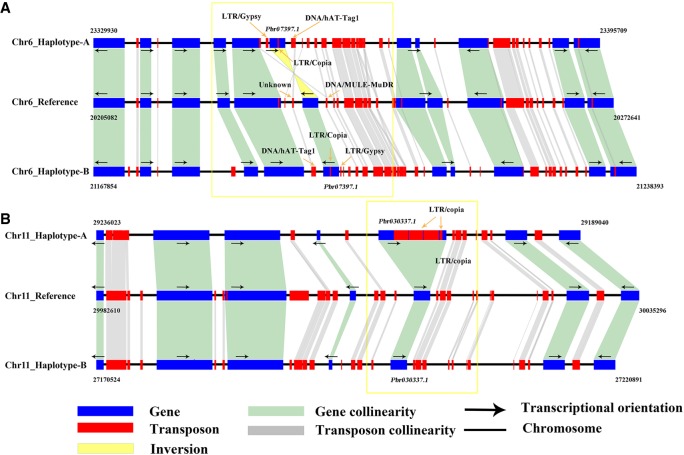
The difference in transposon insertions between haplotype A and haplotype B. (*A*) Inversion insertions around *Pbr020217.2* in a region with high collinearity between the reference, haplotype A, and haplotype B. Orange arrows show the positions of transposons. (*B*) Four LTR/copia transposon insertions in *Pbr030337.1*. Orange arrows show the positions of transposons.

Mutations caused by transposon insertions in or near genes can alter gene expression or the structure of the encoded proteins (Kobayashi et al. 2004; International Peach Genome Initiative et al. 2013), which is a key driving force and important reason for the genetic diversity of many species. In addition, there is ∼271.9 Mb repetitive sequences (53.1%) in the pear reference genome (Wu et al. 2013). To find the large indels between two haplotypes (which is difficult to achieve with the reference genome), we identified some transposon insertions in a region with high collinearity among the reference, haplotype A, and haplotype B genomes. As shown in Figure 4A, LTR/copia elements were inserted inside *Pbr007397.1*, and LTR/Gypsy/hAT-Tag1 elements were inserted around *Pbr007397.1* as well as around the inversion between haplotype A and haplotype B. As shown in Figure 4B, four LTR/copias were inserted inside *Pbr030337.1* only in haplotype A, whereas there is no LTR insertion in haplotype B. This suggests that the structural difference between the two haplotypes in pear can be distinguished by 12 pollen cells, and it will improve the research in the potential functional roles of transposon in pear genome.

Having the phased haploid genome assemblies also facilitates genetic studies. Pear is known to harbor a gametophytic self-incompatibility (GSI) that is controlled by an apparently single, multiallelic locus (the S-locus) that contains a pistil S-determinant (*S-RNase*) and a pollen S-determinant (*SFB*) (de Nettancourt 1997). In our new assemblies, the *S_17_-RNase* gene was anchored 900 kb from the end of haploid genome B linkage group 17 (LG 17), and six candidate *SFB* genes were anchored in tandem ∼3.9–4.0 M from the end of haploid genome B LG17. Therefore, the physical location of the *S-RNase* gene and the six candidate *SFB* genes in haploid genome B is at the end region of LG17, from 0.9 to 3.9 M, which is consistent with its location on a genetic map (Yamamoto et al. 2007). Thus, using phased haploid genome assemblies enables a more accurate determination of the genomic position of the S-locus.

### Allele-specific expression from the A and B haploid genomes

In diploid organisms, expression of both alleles is a complex trait affected by various factors. Although microarray and allele-specific RT-PCR analysis can readily distinguish the expression of different alleles of some genes (Tarutani and Takayama 2011; Reinius and Sandberg 2015), it may be challenging to phase longer genes. We identified 29,465 and 28,984 allelic genes in haploid genome A and haploid genome B, respectively, with 236 alleles present only in haploid genome A and 291 allelic genes only present in haploid genome B. Given the present ubiquity of RNA-seq methods in functional genomics research, having phased haploid genome sequences can readily facilitate distinguishing any allelic diversity in the expression patterns of a given gene. We next reanalyzed RNA-seq data for samples from four stages of pear fruit development (falling stage, swelling stage, later swelling stage, and ripeness stage) in light of our newly phased haploid genome assemblies to identify the potential differential functions of allelic genes in pear fruit development and physiology.

**Figure 5.**
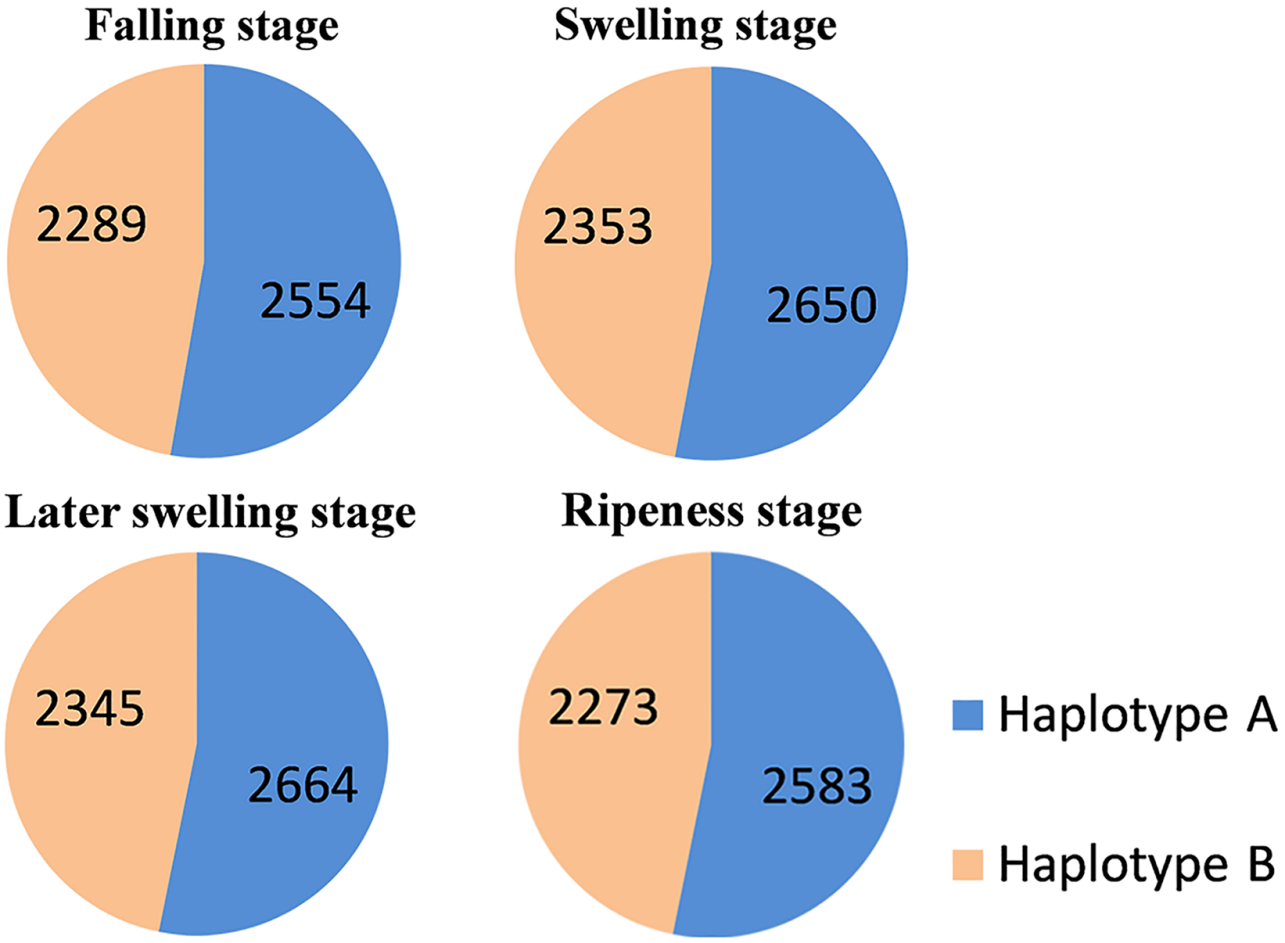
Monoallelic expression in the development of pear fruit. The four stages include the falling stage, swelling stage, later swelling stage, and ripeness stage. Blue shows the number of monoallelic expression in haplotype A. Orange shows the number of monoallelic expression in haplotype B.

We found 1926 genes with differentially expressed alleles and 2079 with monoallelic expression (i.e., in which only one allele was expressed) (Fig. 5). KEGG enrichment analysis of the genes with differential allelic expression indicated a tendency for such genes to be associated with pathways including “Biosynthesis of secondary metabolites,” “Flavonoid biosynthesis,” and “Terpenoid backbone biosynthesis” (Supplemental Table S9). Three of the major economically important fruit quality traits in pears are sugar content, volatile profiles, and the extent of so-called “stone cells.” Hence, we also identified 95 genes of the 4005 allelic genes with differential allelic expression related to these trains (Supplemental Fig. S1–S3). Overall, our results suggest that allelic expression is substantially involved in controlling the development of pear fruit traits, so our assembly of haplotype-resolved genomes adds resolution that will allow faster identification of economically important genes in pear.

### Meiotic recombination in 12 pollen cells

**Figure 6.**
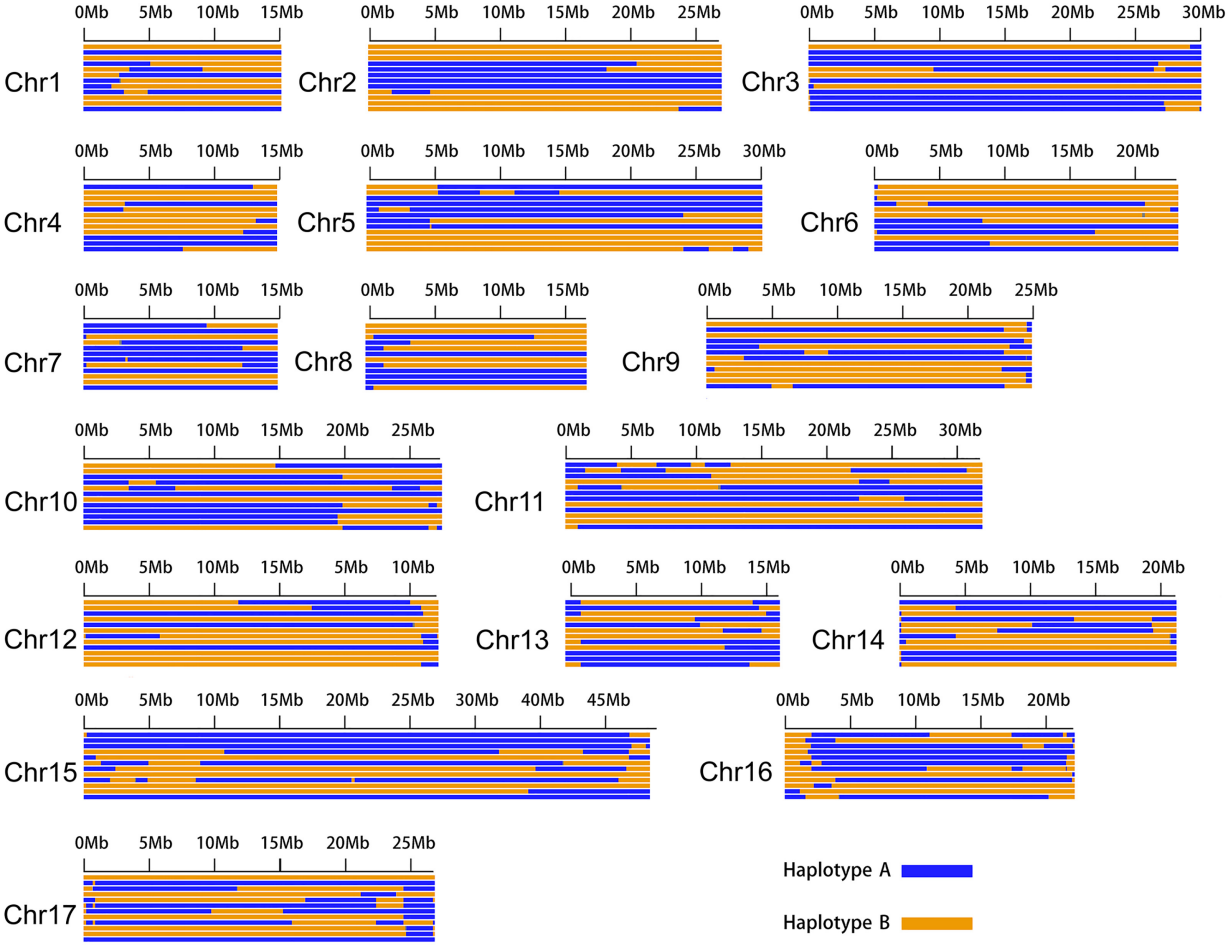
Meiotic recombination in 12 pollen cells. Haplotype A and haplotype B are shown in different colors, with switching points between the haplotypes representing meiotic crossover (MCO) events.

The majority of eukaryotes reproduce via the meiotic cell division during prophase I, in which chromosome double-strand breaks are initiated and repaired by homologous recombination, resulting in genomic exchanges (meiotic crossover, MCO) (Ziolkowski et al. 2017). The location of MCO events was identified to construct a recombination map for 12 pollen cells. The genotype of each cell was compared among the 17 haplotype A chromosomes. For any given pollen cell, evidence for a MCO was confirmed by a switch between identity or nonidentity. The 12 pollen genomes with the largest number of genotype calls were characterized, and 264 MCO events were identified, with an average of 1.3 events per chromosome (Fig. 6). This is considerably lower than the 1.9 MCOs observed per maize chromosome (Li et al. 2015) and higher than the 0.9 MCOs observed per *Arabidopsis* chromosome (Lu et al. 2012a). Additionally, we found that the “12-bit binary code” used in BAC phasing revealed a similar pattern of MCO events in the 12 pollen cells (Fig. 6; Supplemental Table S3). To evaluate the frequency of MCO events at the genome level, the number of MCOs at each location in the genome was calculated. We found that MCOs mainly occurred at the ends of each pear chromosome (Supplemental Fig. S4), which is consistent with what has been reported in maize (Lynn et al. 2002), yeast (Mancera et al. 2008), and human (Lu et al. 2012b). We did not analyze gene conversions and crossover interference because of the limited number of MCO events, but these findings will be helpful for obtaining a better understanding of meiotic recombination in pear.

## Discussion

Haplotype-resolved genome assemblies offer clear benefit over working with assemblies that have been built from diploid materials that frequently feature mosaic sequences. A popular strategy to generate haploid genome-resolved sequence data is the sampling of gametes, and the advent of single-cell sequencing has further advanced such methods. Single-cell whole-genome amplification technology is conducted frequently in animals (Kirkness et al. 2013; Duan et al. 2018) and is used routinely in cancer research, but has been used far less frequently with plant cells, owing in part to the influence of plant cell walls on the efficiency of amplification. We used an improved protocol for the isolation of protoplasts from pollen cells (Qu et al. 2007) and amplified single-pollen-cell genomic sequences. This enabled us to obtain an average of ∼66.25% breadth of coverage of the pear genome for each of the 12 pollen cells for which we obtain sufficient DNA after the MDA amplification protocol.

In fact, amplification of only one haploid pear pollen genome is the best method for haplotyping, if one could be sequenced completely. However, because of the limitations of single-cell whole-genome amplification techniques in plant cells, it is unlikely that we could obtain a complete haploid genome sequence from one pollen cell, even when sequencing at a higher depth. In addition, the range of breadth of coverage for a single cell was unstable, ranging from 49.55% to 75% (Supplemental Table S2). Variability in breadth of coverage was also observed in a maize microspore study (25.5% to 48.8%) (Li et al. 2015). This suggests that the breadth of coverage rate obtained from ultralow content amplification is correlated with not only the sequencing depth but also amplification efficiency (Hou et al. 2015). Although the breadth of coverage of single-cell sequencing has substantially improved with our protocol compared to previous studies, it also shows that there is a long way to go before sufficient breadth of coverage can be obtained with plant cell whole-genome amplification technology. Furthermore, MDA genome representation appears to be random across different pollen amplification, so the use of independent samples can be complementary to one another and in aggregate will lead to near complete genome representation. Therefore, to increase the breadth of coverage, we used genome sequence data from multiple cells to cover more BAC sequence. Different pollen cells compensate for each other in terms of uneven amplification and missing data, because each extraction is independent. Consistent with this, sequence from all 12 pollen cells collectively covers 98.85% of the genome, whereas the sequence from individual pollen cell only covers 49.55%–77.92% with a median of 66.26%.

Using reproductive cells for haplotyping was first reported for the assembly of a human genome (Kirkness et al. 2013), the genome of Craig Venter. In the human study, seven sperm cells were directly used for haplotyping. The SNP sites in each cell were identified using microarrays, but there was no long-read sequence data for avoiding the effect of homologous recombination. Hence, only a few regions could be resolved, and the final reconstructed haplotype was much less complete than a true haplotype-resolved assembly that has base-level resolution. In our study, 12 reproductive cells were used to guide BAC phasing, and the final haplotype-resolved assemblies were still based on BAC sequence. Thus, the effect of mosaic recombination on the assembly of haplotype-resolved genomes was reduced and more transposon insertions could be identified. Additionally, we found that most MCOs were located at the ends of each pear chromosome, with an average of 1.3 events per chromosome, and the recombination landscape of pear was defined with better precision. Therefore, our method could filter out meiotic events, random SNP sites caused by amplification errors by MDA were also filtered out because MDA sequences were not directly used in the final assembly. Additionally, we suspected whether no-call to “0” could be used for haplotype phasing before this work. Hence, we set a prerequisite: The genotype for a given SNP position on a BAC must occur in at least two pollen cells. After that we found the no-call to “0” is very low. So, we thought there was a little effect on haplotype phase and started this work. Furthermore, we think that a more sophisticated version dealing with the problem of two adjacent SNPs with the same barcode, or the swapping of a small number of bits because of either (1) sequencing errors or (2) a recombination event, may be more useful. For example, some penalty to the number of SNPs that change barcode values, and some penalty for flipping a bit so that SNPs do not change barcodes (e.g., fixing an error) can be used. In addition, a set of bits can be found to flip that minimize barcode swap + error fixing swaps. This is difficult with this framework, but it could correct the possibility of SNP calling error and also generate a confidence level for each inference coefficient of haplotype phase.

Mosaic assembly in many draft genomes results from the merging of heterozygous loci into single “consensus” sequences (Weisenfeld et al. 2017), and the problem of mosaic assembly is particularly severe when using short sequence reads for assembly (Cao et al. 2015; Du et al. 2017). The short read length prevents accurate reconstruction of distinct alleles because of conflicts in assembly paths, especially when there is structural variation between the two alleles. Although long-read sequencing technology was developed to alleviate this problem, the sequences are still not long enough to cross these mosaic areas. In this study, we overcame this by using sequence from haploid pollen cells to guide BAC reads across the mosaic areas to achieve haplotype phasing. However, we also found that the scaffold N50 (108 kb, 107 kb) of the haplotype-resolved pear genome is lower than that of the reference genome (540.8 kb), which was also observed for the haplotype-resolved human genome assembly obtained using fosmids (YH reference N50 = 23,192 kb, haploid-resolved diploid genome N50 = 484 kb) (Cao et al. 2015). We think one of the reasons for lower contiguity may be the MDA products and is the BAC contiguity (N50 = 17.2). Nevertheless, we found that the initial contig N50 was about 2.8–3 times longer than that of the merged assembly after phasing BACs using the sequence data from 12 single pollen cells (Supplemental Table S5). The improved contiguity of the sequences led to much more accurate gene models, as shown by the >5% increase in BUSCO genes (Table 1), as well as a much higher validation rate of the loci selected for Sanger sequencing. In conventional genome assemblies, although heterozygosity affects the assembly quality, the missing genome sequences can be made up using sequence from another haplotype. Some allelic losses between haplotypes are real, and an entire gene can be missed in a collapsed assembly, depending on which allelic path is chosen by the assembly software. Our current study calls for more careful validation and assessment of draft genome assemblies for outcrossing taxa.

Vegetative tissue is developed from a female gametophyte in gymnosperms; hence, it is convenient to obtain a huge amount of haplotype DNA, which is impossible in angiosperms (Neale et al. 2014). Although breeding a haplotype genome for genome assembly could unambiguously solve this problem, it is laborious and the probability of success is relatively low, which is not suitable for all plant species. For instance, decades of breeding efforts were needed to get a DH Golden Delicious apple line (GDDH13) for improving genome assembly (Daccord et al. 2017), and unsuccessful cases of attempts to use anther cultures (Xu et al. 2013) for breeding haplotype material have been reported. However, the pipeline developed here lays the direct foundation for haplotype assembly of genomes in species with high heterozygosity, and should thus find use with many highly heterozygous species like grapes (Jaillon et al. 2007), potato (The Potato Genome Sequencing et al. 2011), and peach (International Peach Genome Initiative et al. 2013), among others.

Although 12 single pollen cells were used for BAC phasing in this study, BAC data is not a prerequisite for our approach. Indeed, we could easily have used any other long-read sequences such as fosmids, single-molecule real-time sequencing, and the Oxford Nanopore sequencing with read lengths up to 1 Mb (https://nanoporetech.com/about-us/news/world-first-continuous-dna-sequence-more-million-bases-achieved-nanopore-sequencing). We could even directly use WGS contigs for haplotype phasing. The longer the length of sequence reads, the more beneficial it is for haplotype phasing to leverage single pollen haplotype information. We could in theory take advantage of the haploid genome information present in plant pollen to assemble phase-resolved haploid genomes, which is essential for understanding allele-specific events and will facilitate studies of epigenetic regulation and high-resolution population genetics at high resolution. Moreover, we believe our method will be valuable as rapid advances in haploid cell genotyping and high-throughput sequencing technology further enable inexpensive chromosome-scale phasing, which will lead to better and more informative reference genomes as well as gene models for many presently unsequenced angiosperm species.

## Methods

### Material preparation

Pollen from pear, *Pyrus bretschneideri Rehd*, was collected in the orchards of Nanjing Agricultural University, Jiangsu, China, and preserved by drying in air at room temperature for 24 h. The dried pollen was then stored in silica gel at −20°C. The culture medium for pollen contained the following components: 1.5 mM H_3_BO_3_, 1.40 mM MgSO_4_, 0.4 mM Ca(NO_3_)_2_, 292 mM sucrose, and 5 mM 2-(N-morpholino) ethanesulfonic acid hydrate (MES) at pH 6.0 (adjusted with Tris). The cell lysis enzyme buffer contained the following components: 36% D-sorbitol solution, 0.4% (w/v) macerozyme R-10, 1.0% (w/v) cellulase R-10.

### Isolation and lysis of single pollen cells

Mature pear pollen was incubated in culture medium for 40 min to allow germination and growth. Cell lysis enzyme buffer (3:1) was added into the culture medium for 10 min at 30°C to release the pollen tube protoplasts, which were then pipetted onto a glass slide. After that, a Flaming/Brown Micropipette Puller (Sutter Instrument Company) was used to obtain a thin glass pipette, and an electric micromanipulator (Sutter Instrument Company) was used to isolate single protoplast cells, which were aspirated into PCR tubes filled with PBS buffer from the TruePrime Single Cell WGA Kit.

### Single-cell DNA whole-genome amplification

A TruePrime Single Cell WGA Kit was used to lyse single pollen cells and amplify single-pollen-cell DNA through multiple displacement amplification (MDA) (Dean et al. 2001) based on the standard protocol. The whole-genome amplification products were purified with AMPure XP beads.

### Quality control of single-cell amplification products and whole-genome sequencing

Eleven polymorphic molecular markers were designed based on the reference genome sequence (Wu et al. 2013). Low-quality DNA samples with abnormal or undetectable segregation in more than three of the 11 markers were discarded. A total of 12 single-cell whole-genome amplification samples were selected for further high-throughput Illumina sequencing. The TruSeq DNA Sample Prep v2 Kit (Illumina) was used to construct Illumina Standard DNA Libraries, and each sample was sequenced on the Illumina HiSeq 2000/4000 platform. In total, ∼1 billion raw reads from 12 single cells were obtained and filtered to exclude reads containing adapters, low-quality sequence, and unknown bases. All the clean reads were mapped to the pear reference genome using BWA (Li and Durbin 2009) to assess the mapping rate and the breadth of coverage rate for each pollen cell.

### BAC phasing and haplotype-resolved genome assembly

First, each BAC was assembled with SOAPdenovo2 (Luo et al. 2012) with *K* = 27. Second, we aligned sequencing data from 12 single cells to each assembled BAC with BWA (Li and Durbin 2009), and based on the alignment results, SNPs were called with GATK (Van der Auwera et al. 2013). Each assembled BAC was aligned to the chromosome sequences of the reference genome with BLAST (Boratyn et al. 2013). If the breadth of coverage of the BAC sequence on one chromosome reached a minimum of 80%, we assigned the BAC to that chromosome. There are 17 chromosomes in the pear genome, so we could assign the BACs to 17 groups. Third, we used the pattern of the BACs assigned to each chromosome to assign BACs to each haplotype chromosome using an in-house Perl script (for details, see Supplemental Material). For each BAC, we divided the 12 single microspores into two groups based on SNP genotype. We used a 12-bit binary barcode to represent the relationship between each BAC and the 12 single pollen cells, in which each bit represents a single pollen cell. If most SNP sites in the single pollen cell had the same genotype as a BAC, the corresponding bit was assigned a value of “1,” otherwise the value was “0.” The distance between two barcodes was calculated with exclusive disjunction, and then the number of “1” bits was calculated. We used the frequencies of each barcode to calculate the distance of each unclassified BAC to each chromosome. Each haplotype chromosome was assembled using SOAPdenovo2, with the sequencing data for the corresponding BACs and the unclassified BACs. WGS mate-pair sequencing data were used to build superscaffolds.

### Allelic gene annotation and identification of mosaic genes in the reference genome

Based on the annotated genes in the pear reference genome, genes in the two haplotype-resolved genomes were reannotated using GMAP (version 2017-10-12) (Wu and Watanabe 2005), and the breadth of coverage of each gene <70% and gene identity <90% was filtered out. Then genes annotated by GMAP were predicted again using exonerate (Slater and Birney 2005) to confirm the annotations. Protein sequences, coding sequences, and mRNA sequences, including the introns, from the reference and two haplotype genomes were used for multiple sequence alignments with MUSCLE (Edgar 2004) to identify mosaic genes in the reference genome, which is totally identical to haplotype A or haplotype B and partially identical to the reference genome.

### Crossover analysis at the genome level

Clean data from each pollen cell were aligned to the haplotype A genome using BWA (Li and Durbin 2009). SAMtools were used for SNP calling (Li and Durbin 2009). The genotypes of the haplotype A genomes from each pollen cell were compared, and meiotic crossover (MCO) events were defined based on a switch between identity or nonidentity to the haplotype A genome.

### Allele-specific expression and pathway analysis

RNA-seq reads were aligned to haplotype A and haplotype B genome by Bowtie 2 (Langmead and Salzberg 2012). Quantification of allele-specific expression was performed using the reads per kb per million reads (RPKM) method (Mortazavi et al. 2008).

KEGG is a highly integrated database for systematic analysis of gene functions in terms of the networks of genes and molecules (http://www.genome.jp/kegg/). KEGG pathway analysis was performed to identify pathways significantly enriched in genes with differentially expressed alleles. Pathways with significant enrichment scores (*P* < 0.05 and FDR < 0.05) were defined as significant pathways (Yi et al. 2006; Kanehisa et al. 2007).

## Data access

The high-throughput sequencing data from this study have been submitted to the NCBI BioProject database (https://www.ncbi.nlm.nih.gov/bioproject/) under accession numbers PRJNA554374 and PRJNA563942. All scripts generated in this study are available as Supplemental Code.

## Supplementary Material

Supplemental Material
