# Supplementary material for: Single-pollen-cell sequencing for gamete-based phased diploid genome assembly in plants
Source: Genome Res. 2019 Nov;29(11):1889–99. doi: 10.1101/gr.251033.119 (PMC6836740; doi:10.1101/gr.251033.119)
Supplement: Supplemental Material [file supp_29_11_1889__index.html]

Single-pollen-cell sequencing for gamete-based phased diploid genome assembly in plants — Supplemental Material 

# Single-pollen-cell sequencing for gamete-based phased diploid genome assembly in plants

## Supplemental Material

- Supplemental\_Material.docx
- Supplemental\_Table\_S10.xlsx
