# Supplementary material for: Single-pollen-cell sequencing for gamete-based phased diploid genome assembly in plants
Source: Genome Res. 2019 Nov;29(11):1889–99. doi: 10.1101/gr.251033.119 (PMC6836740; doi:10.1101/gr.251033.119)
Supplement: Supplemental Material [file supp_gr.251033.119_Supplemental_Material.docx]

For *GENOME RESEARCH*

SUPPLEMENTAL INFORMATION

Single-pollen-cell sequencing for gamete-based phased diploid genome assembly in plants

Dongqing Shi^1†^, Jun Wu^1†^, Haibao Tang^2†^, Hao Yin^1†^, Hongtao Wang^3^, Ran Wang^4^, Runze Wang^1^, Ming Qian^1^, Juyou Wu^1^, Kaijie Qi^1^, Zhihua Xie^1^, Zhiwen Wang^5^, Xiang Zhao^5^, Shaoling Zhang^1*^

^1^Centre of Pear Engineering Technology Research, State Key Laboratory of Crop Genetics and Germplasm Enhancement, Nanjing Agricultural University, Nanjing 210095, China; ^2^Center for Genomics and Biotechnology, Fujian Agriculture and Forestry University, Fuzhou, Fujian Province, China; 3School of Life Science, Henan University, Kaifeng, 475004, China; ^4^College of Agriculture, Qingdao Agricultural University, Qingdao, 266109, China; ^5^ PubBio-Tech, Wuhan, 430070, China;

†Dongqing Shi, Jun Wu, Haibao Tang, Hao Yin contributed equally to this work.

*Correspondence author: Shaoling Zhang: Tel.: +86-25-84396580; Fax: +86-25-84396485; E-mail: slzhang@njau.edu.cn

**Supplemental Information**

**Table of Contents**

**Supplemental Note**

**Supplemental_Table_1 to 9**

**Supplemental_Fig_1 to 4**

**Supplemental Code_Script_1 to 3**

**Supplemental_Table_S1. Primers used to estimate the integrity of the amplified DNA product**

| **Name** | **Left primer** | **Right primer** | **Product size** |
| --- | --- | --- | --- |
| Chr2 | GTCGCCAAACAACTCATTACTCTG | CAACCAACACCCAAATATCTCCC | 485 |
| Chr3 | GAGGGATGAAGGCTGTGACGATG | CGGAACTCACCTGGGTAAAGACA | 494 |
| Chr4 | TCTTGGAAATATGCCCGAGAAT | ATAACGCCACTGCTTGTAACCC | 222 |
| Chr5 | CTCTTCTTTCGTGGCTCTGCT | TCGTACCTGTCCATTTGGGAT | 133 |
| Chr6 | GCTGATGCCAACTATGGGATTAT | CAAACTGGGAAACAAATACGATACA | 166 |
| Chr7 | TCGTAACTGCAAGTTTGAGGGAG | TTGAAGTGGTGGGTGTCAAGATC | 222 |
| Chr8 | ATTCAAGGCGTTTGGAGTATGTC | TTCTTGGAATCAACTGGACCATC | 451 |
| Chr11 | ATCGAGTTCCAACTGAAGAAGCA | AATCGACCAATTTCGCCATAGAC | 268 |
| Chr12 | TAGTGATGTTAGTGGCGGTGGG | AACGTGCTCTTACAACTGATCTCCT | 211 |
| Chr15 | CTGTAATGCTTGGCCCGATTGT | GAGCGAGCCCTTCCACGGTA | 250 |
| Chr16 | AGGTTAGAGTTCGTGGGCGTTGT | ACGAGTGGTCACCCTCCGTAAA | 190 |

**Supplemental_Table_S2. Pollen sequence data statistics**

| **Cell number** | **Total Reads** | **Total Mapped Reads** | **Mapped Rate (%) & rate of unique loci (%)** | **Breadth of Reference Genome Coverage (%)** | **Depth of Reference Genome Coverage (%)** | **SNP on reference genome** |
| --- | --- | --- | --- | --- | --- | --- |
| 1 | 209873047 | 199939018 | 85.27% & 71.36% | 55.05% | 29.86808069 | 1931525 |
| 2 | 62610776 | 53132454 | 84.86% & 83.99% | 57.86% | 12.88899976 | 1543051 |
| 3 | 74869517 | 59707626 | 79.75% & 79.52% | 61.70% | 12.59794981 | 1464239 |
| 4 | 78640853 | 64534614 | 82.06% & 81.84% | 73.72% | 11.71937661 | 1940265 |
| 5 | 88521594 | 70975037 | 80.18% & 81.00% | 64.29% | 11.19177225 | 1707112 |
| 6 | 82348153 | 66392071 | 80.62% & 81.08% | 66.31% | 10.6550546 | 1236549 |
| 7 | 90566705 | 74100910 | 81.82% & 81.28% | 49.55% | 9.164286148 | 1623207 |
| 8 | 64394384 | 47512585 | 73.78% & 82.94% | 69.46% | 8.910451992 | 1493229 |
| 9 | 60849987 | 47153231 | 77.49% & 83.44% | 71.46% | 8.659865323 | 1480585 |
| 10 | 56551505 | 44282280 | 78.30% & 80.79% | 77.38% | 8.403503795 | 1754957 |
| 11 | 52760695 | 42225070 | 80.03% & 84.05% | 77.92% | 8.048126898 | 1792303 |
| 12 | 59048620 | 43851516 | 74.26% & 83.59% | 70.36% | 7.508637808 | 1548532 |
| All | 981035836 | 813806412 |  | 98.85% | 139.6161057 | 3506724 |
| Average： |  |  | 79.86% & 81.24% | 66.26% |  |  |

**Supplemental_Table_S3. The main 12-bit barcodes for BACs in each haplotype chromosome**

| **Chromosome number** | **12-bit binary code** |
| --- | --- |
| Haplotype-A-Chr1 | 100100100010; 100100110010; 100111001010 |
| Haplotype-B-Chr1 | 011011011101; 011000110101; 011011001101 |
| Haplotype-A-Chr2 | 000011111000; 100011100000; 000011100000 |
| Haplotype-B-Chr2 | 111100000111; 011100011111; 111100011111 |
| Haplotype-A-Chr3 | 000010100001; 000010110001; 110010011010 |
| Haplotype-B-Chr3 | 111101001100; 111101011100; 111101000100 |
| Haplotype-A-Chr4 | 111000010001; 011000001001; 111000001001 |
| Haplotype-B-Chr4 | 000111101110; 100111110110; 000111100110 |
| Haplotype-A-Chr5 | 000010111111; 000010111101; 000010011101 |
| Haplotype-B-Chr5 | 111101000000; 111101000010; 111101100010 |
| Haplotype-A-Chr6 | 100110001000; 100010001000; 110111001000 |
| Haplotype-B-Chr6 | 011101110111; 011001110111; 001000110111 |
| Haplotype-A-Chr7 | 100101111011; 100101110011; 100111101010 |
| Haplotype-B-Chr7 | 011010000100; 011010011100; 111010010101 |
| Haplotype-A-Chr8 | 011100100100; 011110010100; 011100100000 |
| Haplotype-B-Chr8 | 100011010011; 100011101011; 100011011111 |
| Haplotype-A-Chr9 | 100001101010; 100000111010; 100001001010 |
| Haplotype-B-Chr9 | 011110010101; 011010010101; 011110110101 |
| Haplotype-A-Chr10 | 011110101100; 100100101001; 011110111100 |
| Haplotype-B-Chr10 | 100001010011; 100011001011; 100001000011 |
| Haplotype-A-Chr11 | 100101110000; 100101110010; 100100111010 |
| Haplotype-B-Chr11 | 011010001111; 011010001101; 011011000001 |
| Haplotype-A-Chr12 | 000101010100; 000100010100; 000100010111 |
| Haplotype-B-Chr12 | 111011101010; 111010101011; 111011101011 |
| Haplotype-A-Chr13 | 111010010010; 111110101010; 111010001010 |
| Haplotype-B-Chr13 | 000101101101; 000001010101; 100100101110 |
| Haplotype-A-Chr14 | 011000110011; 011000001011; 011001001001 |
| Haplotype-B-Chr14 | 100111001100; 100110000100; 100111110100 |
| Haplotype-A-Chr15 | 100000000111; 100100101111; 110101001111 |
| Haplotype-B-Chr15 | 011111111000; 011011010000; 001010010000 |
| Haplotype-A-Chr16 | 100100111100; 100110100000; 100100111101 |
| Haplotype-B-Chr16 | 011011011111; 011011010111; 111101000011 |
| Haplotype-A-Chr17 | 100101100110; 100101100010; 111111000010 |
| Haplotype-B-Chr17 | 011010011001; 011010011101; 000000111101 |

**Supplemental_Table_S4. The number of phased BACs in each haplotype chromosome**

|  | **Phased in haplotype-A** | **Phased in haplotype-B** |
| --- | --- | --- |
| chr1 | 867(54.05%) | 737(45.95%) |
| chr2 | 967(51.79%) | 900(48.21%) |
| chr3 | 1215(50.94%) | 1170(49.06%) |
| chr4 | 632(45.93%) | 744(54.07%) |
| chr5 | 1058(50.69%) | 1029(49.31%) |
| chr6 | 760(49.32%) | 781(50.68%) |
| chr7 | 645(52.06%) | 594(47.94%) |
| chr8 | 659(45.29%) | 796(54.71%) |
| chr9 | 845(52.65%) | 760(47.35%) |
| chr10 | 918(49.38%) | 941(50.62%) |
| chr11 | 1214(53.34%) | 1062(46.66%) |
| chr12 | 1088(51.22%) | 1036(48.78%) |
| chr13 | 758(46.47%) | 873(53.53%) |
| chr14 | 760(45.32%) | 917(54.68%) |
| chr15 | 1667(53.86%) | 1428(46.14%) |
| chr16 | 819(50.87%) | 791(49.13%) |
| chr17 | 1061(56.41%) | 820(43.59%) |
| Total | 15933(50.88%) | 15379(49.12%) |

**Supplemental_Table_S5. Initial contig N50 statisticsforthe haplotype-A, haplotype-B and merged haplotype-A and haplotype-B chromosomes**

|  |  | **Contig N50 (bp)** | **Contig length (bp)** | **Merged contig N50 (bp)** | **Merged contig length (bp)** |
| --- | --- | --- | --- | --- | --- |
| Chr1 | haplotype-A | 1,570 | 39,087,176 | 681 | 64,862,996 |
|  | haplotype-B | 2,370 | 31,519,528 |  |  |
| Chr2 | haplotype-A | 2,050 | 36,092,530 | 741 | 59,525,396 |
|  | haplotype-B | 1,919 | 34,907,969 |  |  |
| Chr3 | haplotype-A | 1,301 | 43,325,092 | 561 | 70,347,464 |
|  | haplotype-B | 1,682 | 41,670,998 |  |  |
| Chr4 | haplotype-A | 1,503 | 26,185,405 | 633 | 48,580,556 |
|  | haplotype-B | 1,638 | 29,756,602 |  |  |
| Chr5 | haplotype-A | 1,903 | 39,109,596 | 552 | 64,792,190 |
|  | haplotype-B | 1,750 | 37,681,503 |  |  |
| Chr6 | haplotype-A | 1,676 | 29,523,785 | 548 | 47,078,770 |
|  | haplotype-B | 2,095 | 27,829,726 |  |  |
| Chr7 | haplotype-A | 1,760 | 23,319,962 | 539 | 37,186,438 |
|  | haplotype-B | 1,706 | 20,455,730 |  |  |
| Chr8 | haplotype-A | 1,981 | 25,162,150 | 647 | 46,641,571 |
|  | haplotype-B | 1,807 | 29,102,599 |  |  |
| Chr9 | haplotype-A | 1,808 | 32,550,460 | 652 | 51,122,982 |
|  | haplotype-B | 2,041 | 29,158,638 |  |  |
| Chr10 | haplotype-A | 1,926 | 35,206,697 | 504 | 60,831,684 |
|  | haplotype-B | 2,018 | 35,210,549 |  |  |
| Chr11 | haplotype-A | 1,457 | 47,220,581 | 498 | 76,743,179 |
|  | haplotype-B | 1,358 | 42,555,622 |  |  |
| Chr12 | haplotype-A | 1,538 | 42,643,846 | 536 | 71,075,401 |
|  | haplotype-B | 1,542 | 39,092,343 |  |  |
| Chr13 | haplotype-A | 2,086 | 31,592,479 | 833 | 58,887,619 |
|  | haplotype-B | 2,024 | 36,698,995 |  |  |
| Chr14 | haplotype-A | 1,877 | 29,411,654 | 530 | 55,930,761 |
|  | haplotype-B | 1,643 | 35,940,931 |  |  |
| Chr15 | haplotype-A | 1,751 | 54,341,976 | 532 | 98,124,168 |
|  | haplotype-B | 2,009 | 54,132,185 |  |  |
| Chr16 | haplotype-A | 1,593 | 31,010,142 | 516 | 52,279,741 |
|  | haplotype-B | 1,796 | 30,711,262 |  |  |
| Chr17 | haplotype-A | 1,117 | 40,898,043 | 544 | 62,564,228 |
|  | haplotype-B | 1,328 | 35,285,288 |  |  |
| Average | haplotype-A | 1699.823529 |  | 591 |  |
|  | haplotype-B | 1807.411765 |  |  |  |

**Supplemental_Table S6. Genome assembly statistics for haplotype-A and haplotype-B**

|  |  | **Contig N50 (bp)** | **Number of**  **contigs (bp)** | **Contig**  **length (bp)** | **Scaffold**  **N50 (bp)** | **Number of**  **scaffolds (bp)** | **Scaffold**  **length (bp)** |
| --- | --- | --- | --- | --- | --- | --- | --- |
| Chr1 | haplotype-A | 26,033 | 2,764 | 30,866,015 | 111,516 | 583 | 34,633,473 |
|  | haplotype-B | 29,569 | 2,003 | 26,380,803 | 113,003 | 407 | 29,486,347 |
| Chr2 | haplotype-A | 29,823 | 2,483 | 30,524,991 | 120,149 | 575 | 33,982,089 |
|  | haplotype-B | 23,885 | 3,621 | 28,863,276 | 106,980 | 630 | 32,857,736 |
| Chr3 | haplotype-A | 22,736 | 4,509 | 34,216,271 | 102,855 | 865 | 39,000,284 |
|  | haplotype-B | 22,496 | 4,375 | 33,052,831 | 104,400 | 746 | 37,445,476 |
| Chr4 | haplotype-A | 26,383 | 1,897 | 19,841,438 | 93,576 | 461 | 22,481,005 |
|  | haplotype-B | 26,889 | 1,956 | 23,046,958 | 97,934 | 548 | 26,020,322 |
| Chr5 | haplotype-A | 31,383 | 2,720 | 32,817,553 | 111,276 | 719 | 36,232,552 |
|  | haplotype-B | 30,161 | 2,631 | 30,886,156 | 108,974 | 721 | 34,263,553 |
| Chr6 | haplotype-A | 30,563 | 1,997 | 23,542,633 | 108,735 | 635 | 26,156,102 |
|  | haplotype-B | 36,284 | 1,695 | 22,527,059 | 115,182 | 486 | 24,728,010 |
| Chr7 | haplotype-A | 32,659 | 1,453 | 18,276,503 | 108,507 | 332 | 19,988,023 |
|  | haplotype-B | 32,159 | 1,233 | 15,721,596 | 118,228 | 334 | 17,279,375 |
| Chr8 | haplotype-A | 29,331 | 1,754 | 20,775,602 | 105,800 | 427 | 22,876,036 |
|  | haplotype-B | 30,042 | 1,910 | 23,025,870 | 115,930 | 482 | 25,629,564 |
| Chr9 | haplotype-A | 28,149 | 2,406 | 27,345,971 | 117,327 | 592 | 30,346,189 |
|  | haplotype-B | 30,960 | 2,023 | 24,650,087 | 115,863 | 490 | 27,418,332 |
| Chr10 | haplotype-A | 30,596 | 2,480 | 29,136,790 | 118,775 | 640 | 32,508,663 |
|  | haplotype-B | 31,400 | 2,400 | 29,446,047 | 110,242 | 674 | 32,798,812 |
| Chr11 | haplotype-A | 26,422 | 3,395 | 37,294,282 | 103,221 | 914 | 42,481,261 |
|  | haplotype-B | 25,851 | 3,226 | 33,698,692 | 97,948 | 845 | 37,943,032 |
| Chr12 | haplotype-A | 26,607 | 3,059 | 33,936,963 | 97,454 | 896 | 38,128,597 |
|  | haplotype-B | 27,275 | 2,817 | 30,643,450 | 97,934 | 726 | 34,420,915 |
| Chr13 | haplotype-A | 29,331 | 2,085 | 26,207,320 | 105,180 | 485 | 28,678,510 |
|  | haplotype-B | 29,638 | 2,479 | 30,517,347 | 104,795 | 612 | 33,639,845 |
| Chr14 | haplotype-A | 28,466 | 2,065 | 23,825,045 | 114,092 | 522 | 26,563,286 |
|  | haplotype-B | 25,778 | 2,695 | 29,083,733 | 107,309 | 665 | 32,459,367 |
| Chr15 | haplotype-A | 28,355 | 3,904 | 45,061,347 | 108,387 | 1,137 | 49,892,047 |
|  | haplotype-B | 32,123 | 3,735 | 45,425,418 | 121,513 | 920 | 50,678,454 |
| Chr16 | haplotype-A | 28,542 | 2,163 | 24,588,552 | 111,687 | 608 | 27,807,187 |
|  | haplotype-B | 29,033 | 2,229 | 25,179,822 | 99,730 | 664 | 27,835,939 |
| Chr17 | haplotype-A | 25,386 | 2,966 | 31,141,671 | 98,016 | 924 | 35,242,443 |
|  | haplotype-B | 24,404 | 2,720 | 28,463,443 | 95,214 | 756 | 31,854,338 |
| Average | haplotype-A | 28280.29412 |  |  | 108032.5294 | 11315 |  |
|  | haplotype-B | 28702.76471 |  |  | 107716.4118 | 10706 |  |

**Supplemental_Table_S7. Genome lengthstatistics for haplotype-A and haplotype-B**

|  | **Length of assembled haplotype-A** | **Length of assembled haplotype-B** | **Length of anchored haplotype-A** | **Percentage of haplotype-A** | **Length of anchored haplotype-B** | **Percentage of haplotype-B** | **Length of anchored haplotype-A** |
| --- | --- | --- | --- | --- | --- | --- | --- |
| Chr1 | 32,508,663 | 29,486,347 | 13,986,295 | 130.81% | 11,681,249 | 109.25% | 10,691,755 |
| Chr2 | 19,988,023 | 32,857,736 | 24,973,274 | 113.01% | 23,353,708 | 105.68% | 22,098,781 |
| Chr3 | 22,481,005 | 37,445,476 | 27,759,006 | 101.34% | 27,588,444 | 100.72% | 27,392,285 |
| Chr4 | 34,633,473 | 26,020,322 | 13,656,068 | 102.03% | 15,681,159 | 117.16% | 13,384,095 |
| Chr5 | 30,346,189 | 34,263,553 | 27,953,737 | 98.28% | 26,736,942 | 94.00% | 28,442,882 |
| Chr6 | 33,982,089 | 24,728,010 | 21,468,983 | 92.89% | 20,175,727 | 87.30% | 23,112,003 |
| Chr7 | 42,481,261 | 17,279,375 | 13,726,454 | 89.91% | 13,122,132 | 85.95% | 15,267,112 |
| Chr8 | 28,678,510 | 25,629,564 | 15,627,532 | 91.33% | 18,254,227 | 106.68% | 17,110,699 |
| Chr9 | 22,876,036 | 27,418,332 | 22,991,020 | 102.51% | 20,496,897 | 91.39% | 22,428,363 |
| Chr10 | 26,563,286 | 32,798,812 | 25,319,257 | 96.56% | 25,119,908 | 95.80% | 26,220,497 |
| Chr11 | 49,892,047 | 37,943,032 | 29,458,971 | 97.17% | 27,444,205 | 90.53% | 30,316,187 |
| Chr12 | 39,000,284 | 34,420,915 | 25,059,802 | 110.12% | 22,712,876 | 99.81% | 22,757,174 |
| Chr13 | 27,807,187 | 33,639,845 | 15,146,875 | 99.99% | 17,495,662 | 115.50% | 15,147,870 |
| Chr14 | 26,156,102 | 32,459,367 | 19,586,030 | 96.66% | 21,049,062 | 103.88% | 20,263,496 |
| Chr15 | 38,128,597 | 50,678,454 | 40,712,237 | 93.43% | 39,620,623 | 90.93% | 43,574,056 |
| Chr16 | 35,242,443 | 27,835,939 | 20,477,089 | 99.17% | 20,860,707 | 101.02% | 20,649,150 |
| Chr17 | 36,232,552 | 31,854,338 | 24,790,451 | 97.86% | 22,636,069 | 89.36% | 25,332,008 |
| Total | 546,997,747 | 536,759,417 | 382,693,081 | 1.007692686 | 374,029,597 | 0.991147766 | 384,188,413 |

* Percentage of haplotype-A: the percentage of the reference genome covered by haplotype-A

* Percentage of haplotype-B: the percentage of the reference genome covered by haplotype-B

**Supplemental_Table_S8. Primers used to validate the identification of mosaic assembly genes in the reference genome**

| **Name** | **Left primer** | **Right primer** |
| --- | --- | --- |
| Pbr017687.1 | CCCCTTCTACCTCCTTCCTCCAAA | AGATAGCAGCGACACTTCATTCTG |
| Pbr002716.1 | TTGTTCCCAGTTCCAGGTTCAGTTC | GATTAAAGAATTCAGTGATCAAGTCCT |
| Pbr008376.1 | TTTCAACAAGGCTTTCAACTCCAC | ATGGTAATAAGCACTCCTATCATACAT |
| Pbr016900.1 | AGAACTGGCTAGGGAAGCTGTGA | AAGAGAGGGTCATAATGTGCATCCC |
| Pbr017186.1 | GAAATCAAGAAGGCGTATCGGAA | GCAATGCAATAAATCCATCTTCCA |
| Pbr022483.1 | TGTAATACGACTCACTATAGGGCGA | GCAATTCTTTTGATTAGTGGGAGGG |
| Pbr024814.1 | TACTCCCTCCCATCTTTCTGTTTG | CTTATGGTTCTATGATTTCTGATTT |
| Pbr042764.1 | TCCTTCAACCAACCAGAGCCA | TATTTATGAAACTCAACACGGGTACA |
| Pbr032139.1 | GGCAACTTCCAATCGTATAAATCTC | CTAGTCATACAGATAAAATCATTCAAT |
| Pbr013631.1 | CCGTCTGCCACAAACTGTCTTTTT | CTCCGTCAACTTTTAAACGGAGACG |
| Pbr027976.1 | GTCGAACTCCAACCCTTCGATTC | CACGAGATATTAGAGATACCTTGAGCTG |
| Pbr000317.1 | GTGGAGCATGCTTATTATAATGTTGTT | GTAGAAAGAAATTTTTTAATGTGGTA |
| Pbr009655.1 | GGCCTGGCTACAGATGACTTACTTA | ACAAGCATGGGGTTTTTCCACTCAGA |
| Pbr007580.1 | GCCGTCTGGTGCAGCCACTG | CCCTAACTTATTGGACCTCTTGTACAA |
| Pbr005811.1 | CGATATTCGTTAAGGTATCGTTTGG | GCGAAGTCTGAATATCTCATGATGCATA |
| Pbr007495.1 | GGGTCCCAGTTGGTGATCCCTT | GCGGTGAAATTCACCATGTTGCT |
| Pbr003484.1 | CAGTCCCATATGCATCACTATTGAA | CGGAAGTGTTTCGTTAAAACTCCTTAAA |
| Pbr000210.1 | GGTCGTTGTTAAAAAGCAAATAAAA | CCTCCAAATAGCGTCCAAACGATTTG |

**Supplemental_Table_S9. KEGG pathway enrichment of genes with differential allelic expression in the development of pear fruit**

| **MapID** | **MapTitle** | **Pvalue** | **AdjustedPv** | **x** | **y** | **n** | **N** | **EnrichDirect** | **GeneIDs** |
| --- | --- | --- | --- | --- | --- | --- | --- | --- | --- |
| map01110 | Biosynthesis of secondary metabolites | 1.35E-08 | 1.43E-06 | 176 | 2533 | 1926 | 42341 | Over | Pbr014737.1 Pbr039695.1 Pbr032022.1 Pbr004655.1 Pbr013178.1 Pbr000841.1 Pbr005027.1 Pbr020454.1 Pbr041710.1 Pbr020886.1 Pbr020457.1 Pbr015322.1 Pbr021683.1 Pbr038089.1 Pbr023471.1 Pbr024791.1 Pbr041711.1 Pbr019533.1 Pbr024116.1 Pbr004479.1 Pbr005279.1 Pbr041370.1 Pbr018707.1 Pbr035872.1 Pbr009545.1 Pbr005130.1 Pbr027320.2 Pbr015525.1 Pbr022422.1 Pbr022425.1 Pbr031791.1 Pbr002232.2 Pbr040244.1 Pbr035287.1 Pbr028217.1 Pbr021235.1 Pbr041976.1 Pbr020450.1 Pbr007102.1 Pbr028395.1 Pbr007947.1 Pbr007113.1 Pbr011433.1 Pbr015321.1 Pbr020107.1 Pbr020110.1 Pbr031355.1 Pbr027987.1 Pbr006002.1 Pbr028609.1 Pbr034488.2 Pbr021238.1 Pbr039378.1 Pbr020924.1 Pbr032338.2 Pbr024118.1 Pbr042026.1 Pbr038082.1 Pbr004482.1 Pbr018960.1 Pbr018958.1 Pbr009724.1 Pbr033545.1 Pbr018959.1 Pbr042607.3 Pbr026810.1 Pbr007115.1 Pbr016278.1 Pbr016902.1 Pbr021957.1 Pbr019549.1 Pbr000218.1 Pbr029627.1 Pbr007086.1 Pbr014280.1 Pbr027228.1 Pbr014028.1 Pbr027916.1 Pbr016899.1 Pbr012174.1 Pbr033192.1 Pbr026969.1 Pbr015211.1 Pbr007571.1 Pbr041892.1 Pbr009048.1 Pbr014416.1 Pbr018429.1 Pbr017314.1 Pbr001104.1 Pbr015320.1 Pbr041883.1 Pbr017795.1 Pbr038086.1 Pbr007489.3 Pbr013138.1 Pbr028891.1 Pbr036711.1 Pbr003365.1 Pbr000572.1 Pbr028605.1 Pbr006010.1 Pbr021222.2 Pbr032166.1 Pbr027911.1 Pbr023101.1 Pbr023151.2 Pbr039728.1 Pbr039379.1 Pbr027321.1 Pbr028606.1 Pbr004478.1 Pbr027912.1 Pbr028396.1 Pbr029984.1 Pbr032638.1 Pbr020624.1 Pbr024790.1 Pbr041369.1 Pbr032845.1 Pbr021417.1 Pbr017107.1 Pbr040568.1 Pbr023079.1 Pbr033544.1 Pbr010637.1 Pbr025067.1 Pbr000224.1 Pbr011574.1 Pbr000177.1 Pbr021428.1 Pbr015206.1 Pbr027212.1 Pbr003676.1 Pbr013140.1 Pbr034480.1 Pbr033641.1 Pbr000736.1 Pbr019411.1 Pbr005433.1 Pbr006452.1 Pbr027200.1 Pbr040238.1 Pbr027633.1 Pbr018123.1 Pbr034045.1 Pbr018961.1 Pbr032169.1 Pbr042601.1 Pbr027530.1 Pbr006608.1 Pbr005972.1 Pbr032454.1 Pbr020888.1 Pbr018708.1 Pbr014274.1 Pbr000323.1 Pbr017108.1 Pbr042690.1 Pbr027910.1 Pbr023256.1 Pbr040249.1 Pbr024034.1 Pbr020372.1 Pbr006707.1 Pbr006296.1 Pbr034283.5 Pbr017913.2 Pbr039007.1 Pbr036108.1 Pbr023470.1 Pbr006229.1 Pbr000738.2 Pbr017623.1 Pbr018424.1 Pbr005965.1 |
| map00941 | Flavonoid biosynthesis | 5.17E-05 | 0.005480922 | 26 | 243 | 1926 | 42341 | Over | Pbr020454.1 Pbr020886.1 Pbr020457.1 Pbr019533.1 Pbr009545.1 Pbr022422.1 Pbr022425.1 Pbr020450.1 Pbr000218.1 Pbr014028.1 Pbr027916.1 Pbr041892.1 Pbr018429.1 Pbr013138.1 Pbr027911.1 Pbr027912.1 Pbr040568.1 Pbr010637.1 Pbr013140.1 Pbr033641.1 Pbr006452.1 Pbr032454.1 Pbr020888.1 Pbr027910.1 Pbr023470.1 Pbr018424.1 |
| map00900 | Terpenoid backbone biosynthesis | 0.000156514 | 0.016590462 | 14 | 99 | 1926 | 42341 | Over | Pbr005279.1 Pbr002232.2 Pbr028395.1 Pbr011433.1 Pbr026810.1 Pbr033192.1 Pbr024458.1 Pbr041883.1 Pbr028396.1 Pbr032638.1 Pbr000224.1 Pbr034045.1 Pbr023256.1 Pbr020372.1 |
| map00945 | Stilbenoid, diarylheptanoid and gingerol biosynthesis | 0.000331344 | 0.035122471 | 29 | 318 | 1926 | 42341 | Over | Pbr020886.1 Pbr015322.1 Pbr023471.1 Pbr009545.1 Pbr022422.1 Pbr022425.1 Pbr021235.1 Pbr007113.1 Pbr015321.1 Pbr020107.1 Pbr020110.1 Pbr021238.1 Pbr007115.1 Pbr007086.1 Pbr014028.1 Pbr026969.1 Pbr015211.1 Pbr009048.1 Pbr018429.1 Pbr015320.1 Pbr013138.1 Pbr015206.1 Pbr013140.1 Pbr006452.1 Pbr005972.1 Pbr020888.1 Pbr024034.1 Pbr018424.1 Pbr005965.1 |


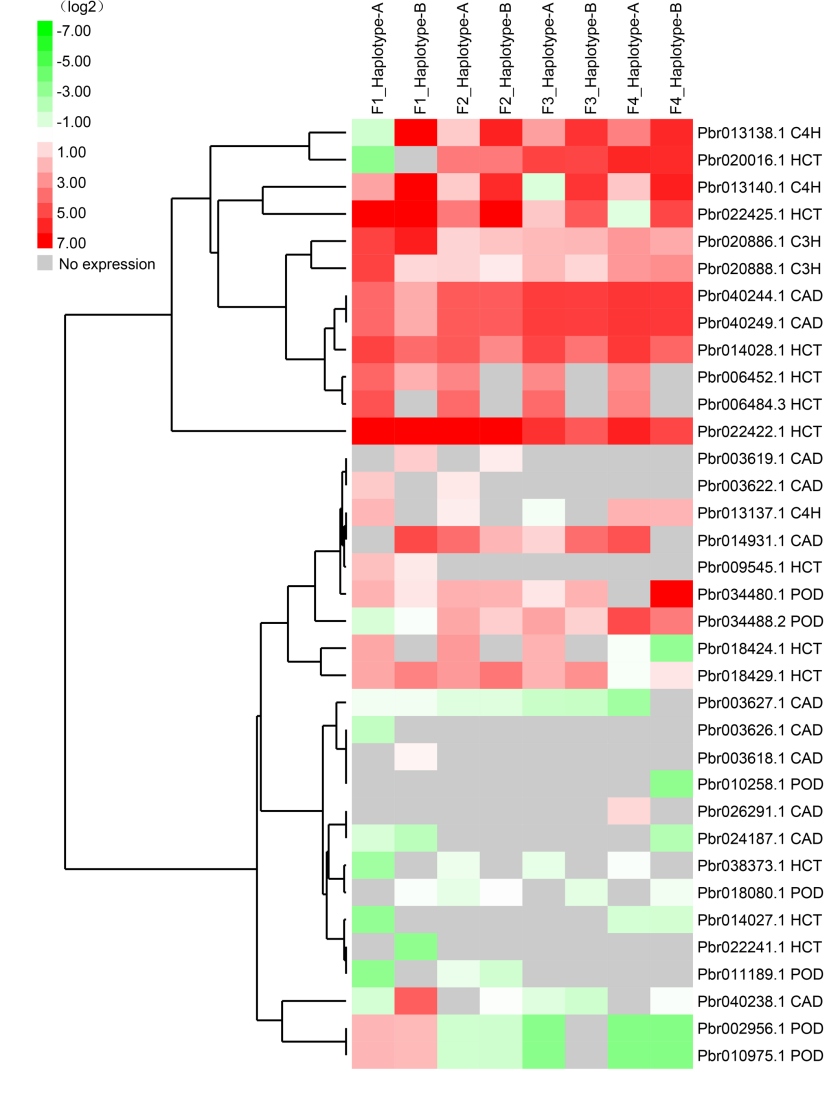


**Supplemental_Fig_S1.Expression pattern of alleles related with stone cell in the development of pear fruit**

F1_Haplotype-A,F2_Haplotype-A,F3_Haplotype-A,F4_Haplotype-A represents four stage of

pear fruit development (falling stage, swelling stage, later swelling stage and ripeness stage) in haplotype-A.

F1_Haplotype-B,F2_Haplotype-B,F3_Haplotype-B,F4_Haplotype-B represents four stage of

pear fruit development (falling stage, swelling stage, later swelling stage and ripeness stage) in haplotype-B.


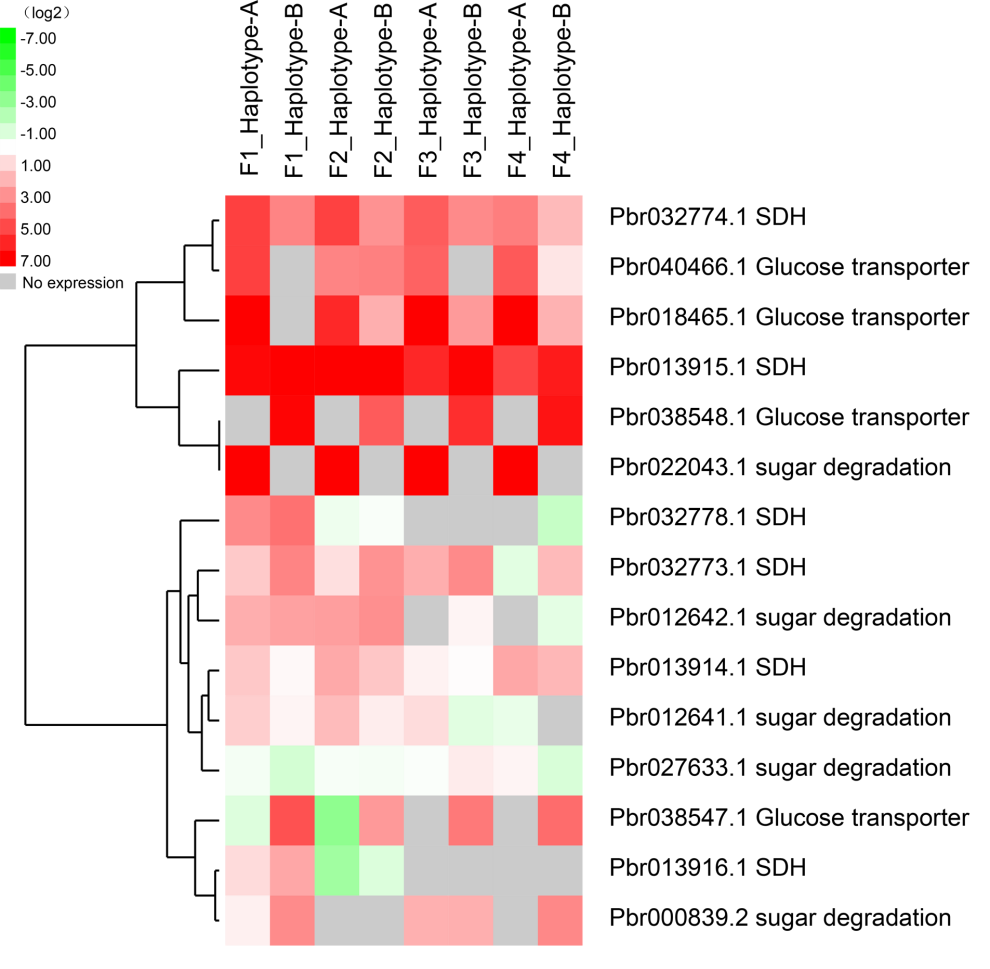


**Supplemental_Fig_S2.Expression pattern of alleles related with sugar in the development of pear fruit**

F1_Haplotype-A,F2_Haplotype-A,F3_Haplotype-A,F4_Haplotype-A represents four stage of

pear fruit development (falling stage, swelling stage, later swelling stage and ripeness stage) in haplotype-A.

F1_Haplotype-B,F2_Haplotype-B,F3_Haplotype-B,F4_Haplotype-B represents four stage of

pear fruit development (falling stage, swelling stage, later swelling stage and ripeness stage) in haplotype-A.


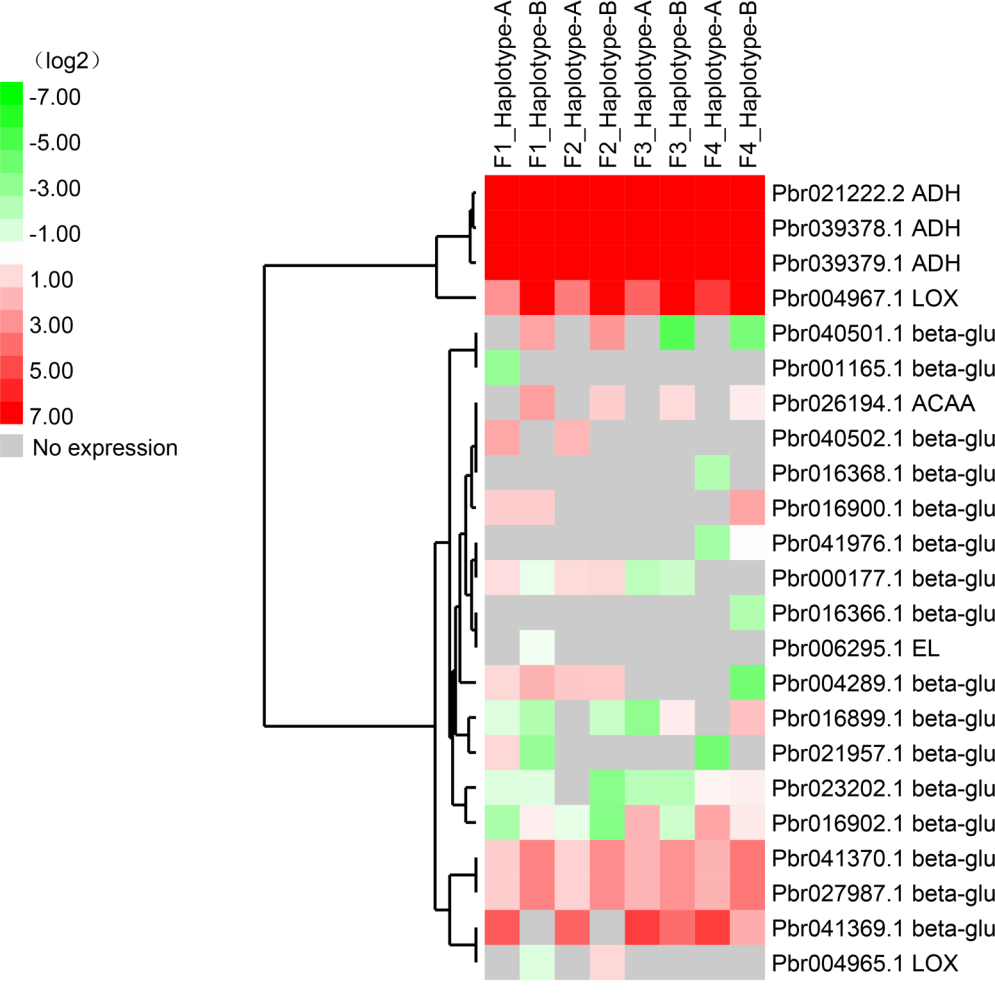


**Supplemental_Fig_S3. Expression pattern of alleles related with volatiles in the development of pear fruit**

F1_Haplotype-A,F2_Haplotype-A,F3_Haplotype-A,F4_Haplotype-A represents four stage

of pear fruit development (falling stage, swelling stage, later swelling stage and ripeness stage) in haplotype-A.

F1_Haplotype-B,F2_Haplotype-B,F3_Haplotype-B,F4_Haplotype-B represents four stage

of pear fruit development (falling stage, swelling stage, later swelling stage and ripeness stage) in haplotype-A.


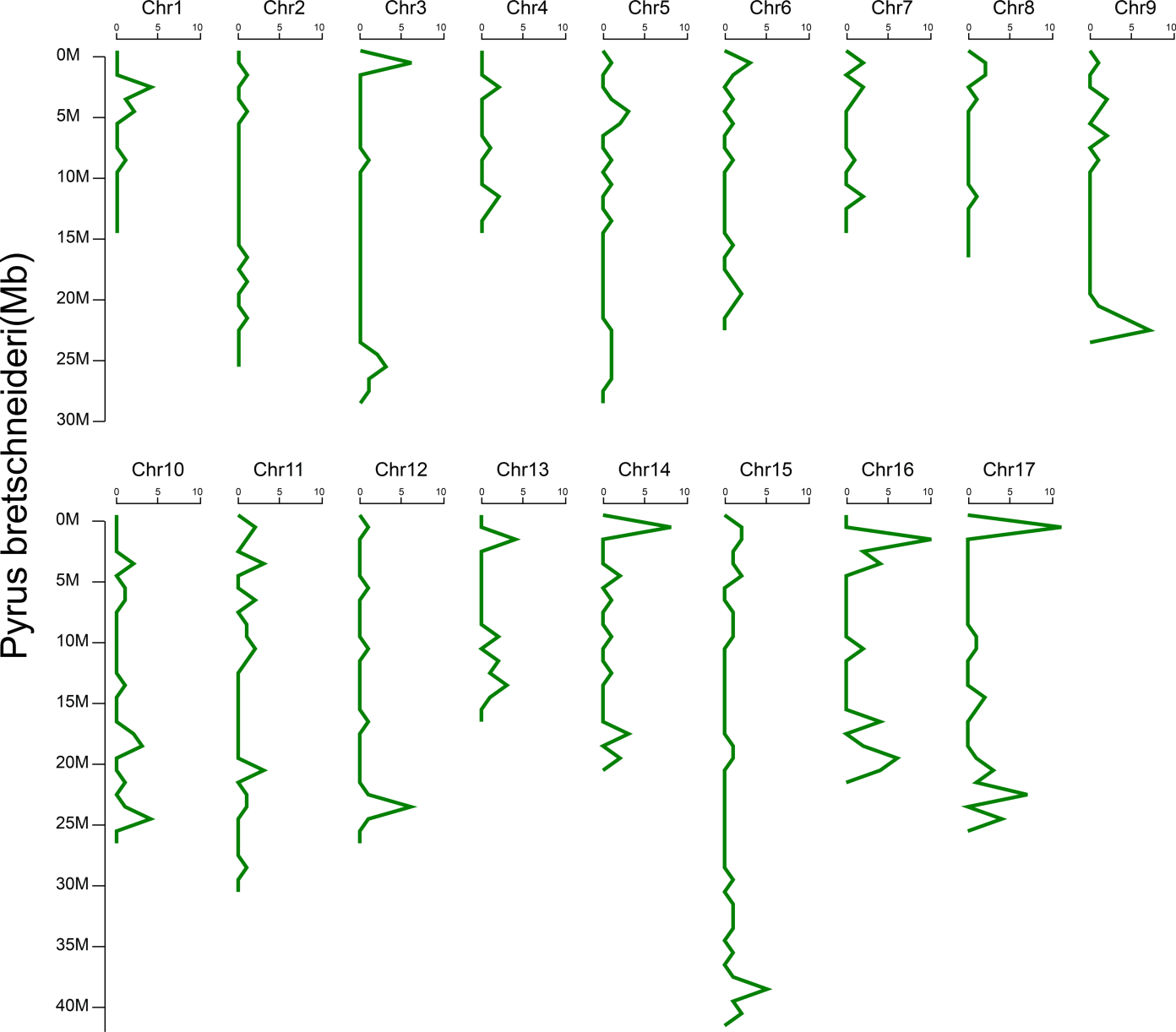


**Supplemental_Fig_S4. The meiotic recombination distribution pattern by using a window size of 1Mb**

**Supplemental Code**

**Script1：This script is to calculate the frequence of each barcode in each chromosome according to BACs which could be mapped to chromosome.**

#!/usr/bin/perl -w

#

#

if($#ARGV != 1){

die "This script is to calculate the frequence of each barcode in each chromosome according to BACs which could be mapped to chromosome.\n Usage: perl $0 <BacMap2Ref.txt><GenotypeFileListofAllBacs> > ChrPattern.txt\n\nBacMap2Ref.txt: This file contains two columns, the first one is BAC name and the second one is corresponding chromosom\nGenotypeFileListofAllBacs: This file lists genotype file of all BACs. The file format of genoetype files is as below:\nCHROM POS REF 1 10_1 10_2 10_5 2 3 4 9_2 9_3 9_4 9_5 9_6 BAC\nscaffold_PAIajnDLIB100-129PIAAPEJ_22_100_0 104 A - - - - - - - - - - - T,T A,A\nscaffold_PAIajnDLIB100-129PIAAPEJ_22_100_0 541 T - T,G - - - - T,G T,G - - G,G T,G T,T\n\nChrPattern.txt: The output files has three columns, the first one is chromosome ID, the sencond is the barcode, and the third is the frequence of the barcode\n";

}

sub classify;

open CHR,"$ARGV[0]";

while(<CHR>){

chomp;

@item=split;

if($item[0]=~/scaffold_[a-zA-Z]+([\d\-]+)\w+_(\d+_\d+)/){

$hash{$1}{$2}=$item[1];

}

}

close CHR;

open LST,"$ARGV[1]";

while(<LST>){

chomp;

if(/scaffoldUnit([\d\-]+)\/(\d+_\d+).snp.txt/){

if(defined($hash{$1}{$2})){

# print $hash{$1}{$2}."\t".classify($_)."\n";

$pattern = classify($_);

$class{$hash{$1}{$2}}{$pattern}++;

}

}

}

close LST;

foreach my $key (keys(%class)){

foreach my $keykey (keys(%{$class{$key}})){

print "$key\t$keykey\t$class{$key}{$keykey}\n";

}

}

sub classify{

open TXT,"$_[0]";

my $name=<TXT>;

my %hash=();

chomp($name);

my @name=split("\t",$name);

for(my $i=3;$i<=$#name;$i++){

if($name[$i] eq "BAC"){

$pos=$i;

}

}

while(<TXT>){

chomp;

my @item=split;

my $num1=0;

my $num2=0;

my %tmp=();

my $flag=0;

for(my $i=3;$i<=15;$i++){

if($item[$i] eq "-"){next;}

if($item[$i] =~ /(\w),(\w)/){

if($1 ne $2){$flag=1;}

}

}

if($flag==0){

for(my $i=3;$i<=15;$i++){

if($item[$i] eq "-"){next;}

if($item[$i] eq "$item[$pos]"){

$num1++;

$tmp{$i}{"same"}++;

}

else{

$num2++;

$tmp{$i}{"diff"}++;

}

}

}

if(($num2>2)&&($num1>2)){

for(my $i=3;$i<=15;$i++){

if(!defined($tmp{$i}{"same"})){$tmp{$i}{"same"}=0;}

if(!defined($tmp{$i}{"diff"})){$tmp{$i}{"diff"}=0;}

$hash{$i}{"same"}+=$tmp{$i}{"same"};

$hash{$i}{"diff"}+=$tmp{$i}{"diff"};

}

}

}

close TXT;

my @same=();my @diff=();

for(my $i=3;$i<=15;$i++){

if($i==$pos){next;}

if(!defined($hash{$i}{"same"})&&!defined($hash{$i}{"diff"})){next;}

if($hash{$i}{"same"}>$hash{$i}{"diff"}){

push @same, $name[$i];

}

else{

push @diff, $name[$i];

}

}

my $same_pattern=0;

if(($#same+$#diff)==10){

for(my $i=0;$i<=$#same;$i++){

if($same[$i] eq "1"){$same_pattern+=2**11;}

if($same[$i] eq "2"){$same_pattern+=2**10;}

if($same[$i] eq "3"){$same_pattern+=2**9;}

if($same[$i] eq "4"){$same_pattern+=2**8;}

if($same[$i]=~/9_2/){$same_pattern+=2**7;}

if($same[$i]=~/9_3/){$same_pattern+=2**6;}

if($same[$i]=~/9_4/){$same_pattern+=2**5;}

if($same[$i]=~/9_5/){$same_pattern+=2**4;}

if($same[$i]=~/9_6/){$same_pattern+=2**3;}

if($same[$i]=~/10_1/){$same_pattern+=2**2;}

if($same[$i]=~/10_2/){$same_pattern+=2**1;}

if($same[$i]=~/10_5/){$same_pattern+=2**0;}

}

}

return $same_pattern;

}

**Script2：This script would classify the unmapped BAC to closest chromosome by calculating distance of barcode between unmapped BAC and each chromosome's barcode pattern.**

#!/usr/bin/perl -w

#

if($#ARGV != 2){

die "This script would classify the unmapped BAC to closest chromosome by calculating distance of barcode between unmapped BAC and each chromosome's barcode pattern.\n Usage: perl $0 <UnMappedBACList><ChrPattern.txt><GenotypeFileListofAllBacs> > UnmappedBAC_distant_Chr.txt\n\nUnMappedBACList: This file contains ID of Unmapped BACs\nChrPattern.txt: This is the output file of Split2chr_S1.pl.\nGenotypeFileListofAllBacs: Descripted in Split2chr_S1.pl.\n\nUnmappedBAC_distant_Chr.txt: There is only 2 column if the distance is the same with each chromosome. There is 4 column if we could find the closest chromosome, the first one is the BAC ID, the second one is barcoe of the BAC, the thired one is chromsome ID, and the last one is the distance. The file format is as below:\n100-129-22_104 all\n100-129-22_108 100010010000 Chr13 3.44943820224719\n100-129-22_111 all\n100-129-22_112 1110101111 Chr4 3.3984375\n100-129-22_113 all\n100-129-22_155 100100100111 Chr1 2\n";

}

#

%classes=();

sub classesify;

open CHR,"$ARGV[0]";

while(<CHR>){

chomp;

@item=split;

if($item[0]=~/scaffold_[a-zA-Z]+([\d\-]+)\w+_(\d+_\d+)/){

$hash{$1}{$2}=1;

$bac_name{$1}{$2}=$item[0];

}

}

close CHR;

open OUT,"$ARGV[1]";

while(<OUT>){

chomp;

@item=split;

if(($item[2]>=20)&&($item[1]!=0)){

$classes{$item[0]}{$item[1]}=$item[2];

}

}

close OUT;

$name="";

open LST,"$ARGV[2]";

while(<LST>){

chomp;

if(/scaffoldUnit([\d\-]+)\/(\d+_\d+).snp.txt/){

if(defined($hash{$1}{$2})){

$name=$1."-".$2;

$hash{$1}{$2}=0;

print "$bac_name{$1}{$2}\t";

$pattern=classesify($_);

if($pattern == 0 ){print "all\n";next;}

print sprintf("%12b",$pattern)."\t";

$chr="";$closest=6;

foreach my $key (keys(%classes)){

$distance{$key}=0;

$num=0;

foreach my $keykey (keys(%{$classes{$key}})){

$xor1=$pattern ^ $keykey;

$dis=0;

$dis1=0;

for($i=0;$i<12;$i++){

$dis1+=($xor1>>$i)&1;

}

$pattern^=4095;

$xor2=$pattern ^ $keykey;

$dis2=0;

for($i=0;$i<12;$i++){

$dis2+=($xor2>>$i)&1;

}

# if(($dis1==0)||($dis2==0)){$distance{$key}=0;last;}

$dis=$dis1<$dis2?$dis1:$dis2;

$distance{$key}+=($dis*$classes{$key}{$keykey});

$num+=$classes{$key}{$keykey};

}

if($num!=0){$distance{$key}/=$num;}

#print "$key\t$distance{$key}\t";

if($distance{$key}<$closest){

$chr=$key;

$closest=$distance{$key};

}

}

print "$chr\t$closest\n";

}

}

}

foreach $key1 (keys(%hash)){

foreach $key2 (keys(%{$hash{$key1}})){

if($hash{$key1}{$key2}==1){

print $key1."-".$key2."\tall\n";

}

}

}

close LST;

sub classesify{

open TXT,"$_[0]";

my $name=<TXT>;

my %hash=();

chomp($name);

my @name=split("\t",$name);

for(my $i=3;$i<=$#name;$i++){

if($name[$i] eq "BAC"){

$pos=$i;

}

}

while(<TXT>){

chomp;

my @item=split;

my $num1=0;

my $num2=0;

my %tmp=();

my $flag=0;

for(my $i=3;$i<=15;$i++){

if($item[$i] eq "-"){next;}

if($item[$i] =~ /(\w),(\w)/){

if($1 ne $2){$flag=1;}

}

}

if($flag==0){

for(my $i=3;$i<=15;$i++){

if($item[$i] eq "-"){next;}

if($item[$i] eq "$item[$pos]"){

$num1++;

$tmp{$i}{"same"}++;

}

else{

$num2++;

$tmp{$i}{"diff"}++;

}

}

}

if(($num2>2)&&($num1>2)){

for(my $i=3;$i<=15;$i++){

if(!defined($tmp{$i}{"same"})){$tmp{$i}{"same"}=0;}

if(!defined($tmp{$i}{"diff"})){$tmp{$i}{"diff"}=0;}

$hash{$i}{"same"}+=$tmp{$i}{"same"};

$hash{$i}{"diff"}+=$tmp{$i}{"diff"};

}

}

}

close TXT;

my @same=();my @diff=();

for(my $i=3;$i<=15;$i++){

if($i==$pos){next;}

if(!defined($hash{$i}{"same"})&&!defined($hash{$i}{"diff"})){next;}

if($hash{$i}{"same"}>=$hash{$i}{"diff"}){

push @same, $name[$i];

}

else{

push @diff, $name[$i];

}

}

my $same_pattern=0;

for(my $i=0;$i<=$#same;$i++){

if($same[$i] eq "1"){$same_pattern+=2**11;}

if($same[$i] eq "2"){$same_pattern+=2**10;}

if($same[$i] eq "3"){$same_pattern+=2**9;}

if($same[$i] eq "4"){$same_pattern+=2**8;}

if($same[$i]=~/9_2/){$same_pattern+=2**7;}

if($same[$i]=~/9_3/){$same_pattern+=2**6;}

if($same[$i]=~/9_4/){$same_pattern+=2**5;}

if($same[$i]=~/9_5/){$same_pattern+=2**4;}

if($same[$i]=~/9_6/){$same_pattern+=2**3;}

if($same[$i]=~/10_1/){$same_pattern+=2**2;}

if($same[$i]=~/10_2/){$same_pattern+=2**1;}

if($same[$i]=~/10_5/){$same_pattern+=2**0;}

}

# my $diff_pattern=0;

# for(my $i=0;$i<=$#diff;$i++){

# if($diff[$i] eq "1"){$diff_pattern+=2**11;}

# if($diff[$i] eq "2"){$diff_pattern+=2**10;}

# if($diff[$i] eq "3"){$diff_pattern+=2**9;}

# if($diff[$i] eq "4"){$diff_pattern+=2**8;}

# if($diff[$i]=~/9_2/){$diff_pattern+=2**7;}

# if($diff[$i]=~/9_3/){$diff_pattern+=2**6;}

# if($diff[$i]=~/9_4/){$diff_pattern+=2**5;}

# if($diff[$i]=~/9_5/){$diff_pattern+=2**4;}

# if($diff[$i]=~/9_6/){$diff_pattern+=2**3;}

# if($diff[$i]=~/10_1/){$diff_pattern+=2**2;}

# if($diff[$i]=~/10_2/){$diff_pattern+=2**1;}

# if($diff[$i]=~/10_5/){$diff_pattern+=2**0;}

# }

#

# my $pattern=$diff_pattern>$same_pattern?$same_pattern:$diff_pattern;

return $same_pattern;

}

**Script3：This script would divide the BACs of each chromosome into two haplotype.**

#!/usr/bin/perl -w

#

if($#ARGV != 4){

die "This script would divide the BACs of each chromosome into two haplotype.\n Usage: perl $0 <BacMap2Ref.txt><Chromsome ID><Chromosome Pattern><UnmappedBAC_distant_Chr.txt><GenotypeFileListofAllBacs> > BACDistanceWithTwoHaplotype.txt\n\nBacMap2Ref.txt: This file contains two columns, the first one is BAC name and the second one is corresponding chromosom\nChromosome ID: Chr1, Chr2...etc.\nChromosome Pattern: This file is generated from ChrPattern.txt. A/B represents two haplotype, the third column is the frequence of the barcode.The file format is as below:\n100100100010 A 99\n11011011101 B 56\n100100110010 A 16\n11000110101 B 15\nUnmappedBAC_distant_Chr.txt: Descripted in Split2chr_S2.pl.\nGenotypeFileListofAllBacs: Descripted in Split2chr_S1.pl\n\nBACDistanceWithTwoHaplotype.txt: The output files has five columns, the first one is BAC ID, A/B represents two haplotype and the following column is the distance vaule.\n";

}

#

%classes=();

sub classesify;

open CHR,"$ARGV[0]";

while(<CHR>){

chomp;

@item=split;

if(($item[1] eq "$ARGV[1]")&&($item[0]=~/scaffold_[a-zA-Z]+([\d\-]+)\w+_(\d+_\d+)/)){

$hash{$1}{$2}=$item[1];

}

}

close CHR;

open OUT,"$ARGV[2]";

while(<OUT>){

chomp;

@item=split;

$item[0]=oct("0b".$item[0]);

$classes{$item[1]}{$item[0]}=1;

}

close OUT;

open LST,"$ARGV[3]";

while(<LST>){

chomp;

if((/scaffoldUnit([\d\-]+)\/(\d+_\d+).snp.txt/)&&(defined($hash{$1}{$2}))){

$name=$1."-".$2;

print "$name\t";

$pattern=classesify($_);

if($pattern == 0 ){print "A\t0\tB\t0\n";next;}

foreach my $key (keys(%classes)){

$distance{$key}=0;

$num=0;

foreach my $keykey (keys(%{$classes{$key}})){

$xor=$pattern ^ $keykey;

$dis=0;

for($i=0;$i<12;$i++){

$dis+=($xor>>$i)&1;

}

$distance{$key}+=$dis;

$num++;

}

if($num!=0){$distance{$key}/=$num;}

print "$key\t$distance{$key}\t";

}

print "\n";

}

}

close LST;

open UNMAP,"$ARGV[4]";

while(<UNMAP>){

chomp;

@item=split;$name=$item[0];

if((defined($item[2]))&&($item[2] eq $ARGV[1])){

print "$name\t";

$pattern=oct("0b".$item[1]);

foreach my $key (keys(%classes)){

$distance{$key}=0;

$num=0;

foreach my $keykey (keys(%{$classes{$key}})){

$xor=$pattern ^ $keykey;

$dis=0;

for($i=0;$i<12;$i++){

$dis+=($xor>>$i)&1;

}

$distance{$key}+=$dis;

$num++;

}

if($num!=0){$distance{$key}/=$num;}

print "$key\t$distance{$key}\t";

}

print "\n";

}

}

close UNMAP;

sub classesify{

open TXT,"$_[0]";

my $name=<TXT>;

my %hash=();

chomp($name);

my @name=split("\t",$name);

for(my $i=3;$i<=$#name;$i++){

if($name[$i] eq "BAC"){

$pos=$i;

}

}

while(<TXT>){

chomp;

my @item=split;

my $num1=0;

my $num2=0;

my %tmp=();

my $flag=0;

for(my $i=3;$i<=15;$i++){

if($item[$i] eq "-"){next;}

if($item[$i] =~ /(\w),(\w)/){

if($1 ne $2){$flag=1;}

}

}

if($flag==0){

for(my $i=3;$i<=15;$i++){

if($item[$i] eq "-"){next;}

if($item[$i] eq "$item[$pos]"){

$num1++;

$tmp{$i}{"same"}++;

}

else{

$num2++;

$tmp{$i}{"diff"}++;

}

}

}

if(($num2>2)&&($num1>2)){

for(my $i=3;$i<=15;$i++){

if(!defined($tmp{$i}{"same"})){$tmp{$i}{"same"}=0;}

if(!defined($tmp{$i}{"diff"})){$tmp{$i}{"diff"}=0;}

$hash{$i}{"same"}+=$tmp{$i}{"same"};

$hash{$i}{"diff"}+=$tmp{$i}{"diff"};

}

}

}

close TXT;

my @same=();my @diff=();

for(my $i=3;$i<=15;$i++){

if($i==$pos){next;}

if(!defined($hash{$i}{"same"})&&!defined($hash{$i}{"diff"})){next;}

if($hash{$i}{"same"}>=$hash{$i}{"diff"}){

push @same, $name[$i];

}

else{

push @diff, $name[$i];

}

}

my $same_pattern=0;

for(my $i=0;$i<=$#same;$i++){

if($same[$i] eq "1"){$same_pattern+=2**11;}

if($same[$i] eq "2"){$same_pattern+=2**10;}

if($same[$i] eq "3"){$same_pattern+=2**9;}

if($same[$i] eq "4"){$same_pattern+=2**8;}

if($same[$i]=~/9_2/){$same_pattern+=2**7;}

if($same[$i]=~/9_3/){$same_pattern+=2**6;}

if($same[$i]=~/9_4/){$same_pattern+=2**5;}

if($same[$i]=~/9_5/){$same_pattern+=2**4;}

if($same[$i]=~/9_6/){$same_pattern+=2**3;}

if($same[$i]=~/10_1/){$same_pattern+=2**2;}

if($same[$i]=~/10_2/){$same_pattern+=2**1;}

if($same[$i]=~/10_5/){$same_pattern+=2**0;}

}

# my $diff_pattern=0;

# for(my $i=0;$i<=$#diff;$i++){

# if($diff[$i] eq "1"){$diff_pattern+=2**11;}

# if($diff[$i] eq "2"){$diff_pattern+=2**10;}

# if($diff[$i] eq "3"){$diff_pattern+=2**9;}

# if($diff[$i] eq "4"){$diff_pattern+=2**8;}

# if($diff[$i]=~/9_2/){$diff_pattern+=2**7;}

# if($diff[$i]=~/9_3/){$diff_pattern+=2**6;}

# if($diff[$i]=~/9_4/){$diff_pattern+=2**5;}

# if($diff[$i]=~/9_5/){$diff_pattern+=2**4;}

# if($diff[$i]=~/9_6/){$diff_pattern+=2**3;}

# if($diff[$i]=~/10_1/){$diff_pattern+=2**2;}

# if($diff[$i]=~/10_2/){$diff_pattern+=2**1;}

# if($diff[$i]=~/10_5/){$diff_pattern+=2**0;}

# }

#

# my $pattern=$diff_pattern>$same_pattern?$same_pattern:$diff_pattern;

return $same_pattern;

}
